# GLP-1 Receptor Agonist Use and Risk of Suicide Death

**DOI:** 10.1001/jamainternmed.2024.4369

**Published:** 2024-09-03

**Authors:** Peter Ueda, Jonas Söderling, Viktor Wintzell, Henrik Svanström, Laura Pazzagli, Björn Eliasson, Mads Melbye, Anders Hviid, Björn Pasternak

**Affiliations:** 1 Division of Clinical Epidemiology, Department of Medicine, Solna, Karolinska Institutet, Stockholm, Sweden; 2 Department of Epidemiology Research, Statens Serum Institut, Copenhagen, Denmark; 3 The Swedish National Diabetes Register, Västra Götalandsregionen, Gothenburg, Sweden; 4 Department of Medicine, Sahlgrenska University Hospital, Gothenburg, Sweden; 5 Pharmacovigilance Research Center, Department of Drug Design and Pharmacology, Faculty of Health and Medical Sciences, University of Copenhagen, Copenhagen, Denmark; 6 HUNT Center for Molecular and Clinical Epidemiology, Department of Public Health and Nursing, Faculty of Medicine and Health Science, Norwegian University of Science and Technology, Trondheim, Norway; 7 Department of Clinical Medicine, Faculty of Health and Medical Sciences, University of Copenhagen, Copenhagen, Denmark; 8 Department of Pediatrics, Stanford University School of Medicine, Stanford, California; 9 Danish Cancer Institute, Copenhagen, Denmark

## Abstract

**Importance:**

Concerns have been raised regarding a link between use of glucagon-like peptide-1 (GLP-1) receptor agonists and increased risk of suicidality and self-harm.

**Objective:**

To assess the association between use of GLP-1 receptor agonists and the risk of suicide death in routine clinical practice.

**Design, Setting, and Participants:**

This active-comparator new-user cohort study used nationwide register data from Sweden and Denmark from 2013 to 2021. Adults 18 to 84 years old who initiated treatment with GLP-1 receptor agonists or the comparator sodium-glucose cotransporter-2 (SGLT2) inhibitors were included. Data were analyzed from March to June 2024.

**Exposure:**

Initiation of treatment with a GLP-1 receptor agonist or SGLT2 inhibitor.

**Main Outcomes and Measures:**

The primary outcome was suicide death recorded in the cause of death registers. Secondary outcomes were the composite of suicide death and nonfatal self-harm and the composite of incident depression and anxiety-related disorders. Using propensity score weighting, hazard ratios (HRs) with 95% CIs were calculated separately in the 2 countries and pooled in a meta-analysis.

**Results:**

In total, 124 517 adults initiated a GLP-1 receptor agonist and 174 036 initiated an SGLT2 inhibitor; among GLP-1 receptor agonist users, the mean (SD) age was 60 (13) years, and 45% were women. During a mean (SD) follow-up of 2.5 (1.7) years, 77 suicide deaths occurred among users of GLP-1 receptor agonists and 71 suicide deaths occurred among users of SGLT2 inhibitors: weighted incidences were 0.23 vs 0.18 events per 1000 person-years (HR, 1.25; 95% CI, 0.83-1.88), with an absolute difference of 0.05 (95% CI, −0.03 to 0.16) events per 1000 person-years. The HR was 0.83 (95% CI, 0.70-0.97) for suicide death and nonfatal self-harm, and the HR was 1.01 (95% CI, 0.97-1.06) for incident depression and anxiety-related disorders.

**Conclusions and Relevance:**

This cohort study, including mostly patients with type 2 diabetes, does not show an association between use of GLP-1 receptor agonists and an increased risk of suicide death, self-harm, or incident depression and anxiety-related disorders. Suicide death among GLP-1 receptor agonist users was rare, and the upper limit of the confidence interval was compatible with an absolute risk increase of no more than 0.16 events per 1000 person-years.

## Key Points

**Question:**

What is the association between use of glucagon-like peptide-1 (GLP-1) receptor agonists and risk of suicide death in patients treated in routine clinical practice?

**Findings:**

In this cohort study of 298 553 adults initiating a GLP-1 receptor agonist or a sodium-glucose cotransporter-2 inhibitor nationwide in Sweden and Denmark, the incidence rate of suicide death was low, and those initiating GLP-1 receptor agonist use did not experience increased risk.

**Meaning:**

This study provides reassuring data showing that those initiating GLP-1 receptor agonist use were not at increased risk of suicide death, although the study could not assess small risk increases.

## Teaser

This cohort study assesses the association between use of glucagon-like peptide-1 receptor agonists and the risk of suicide death in routine clinical practice.

## Introduction

Glucagon-like peptide-1 (GLP-1) receptor agonists are increasingly used in the treatment of type 2 diabetes and obesity. Concerns have been raised regarding suicidality and self-harm linked to use of GLP-1 receptor agonists.^1^ In July 2023, the European Medicines Agency launched an investigation into the safety signal following around 150 spontaneous reports received by the agency regarding suicidal thoughts and thoughts of self-harm potentially associated with the drug. An effect of GLP-1 receptor agonists on suicidality is plausible, as GLP-1 receptors are present in the central nervous system and GLP-1 receptor agonists have been shown to cross the blood-brain barrier.^2,3^ Previous studies have linked bariatric surgery and weight-reduction drugs to a potentially increased risk of suicide and self-harm.^4,5^ Conversely, it has also been suggested that GLP-1 receptor agonists may protect against depression. This hypothesis is based on studies indicating that depression and type 2 diabetes may have partly overlapping causes, including neuroinflammation, and that GLP-1 receptor agonists show neuroprotective properties.^6^

We conducted a cohort study using nationwide data from Sweden and Denmark to examine the association of GLP-1 receptor agonist use with suicide death. In secondary analyses, we assessed the association of GLP-1 receptor agonist use with the composite of suicide and nonfatal self-harm, as well as the composite of incident depression and anxiety-related disorders.

## Methods

### Data Sources

We used health and administrative registers in Sweden and Denmark, including the population registers,^7,8^ prescription drug registers,^9,10^ national patient registers,^11,12^ and cause of death registers,^13,14^ all with nationwide coverage in each country. The registers provide data on demographic variables and vital status, all filled prescriptions from all pharmacies in each country, diagnoses and procedures registered during all outpatient specialist care visits and hospitalizations, and causes of death. Sweden and Denmark have similar universal health care systems and register infrastructure.^15^ Using the same study protocol, we conducted the analyses in Sweden and Denmark separately and performed a meta-analysis of the 2 country-specific estimates.

The study was approved by the Swedish Ethical Review Authority. Informed consent was not needed. Ethical approval is not required for register-based research in Denmark. The study is reported according to the Strengthening the Reporting of Observational Studies in Epidemiology (STROBE) reporting guidelines for cohort studies.

### Study Population

Using an active-comparator new-user design,^16^ we included all new users of either GLP-1 receptor agonists or sodium-glucose cotransporter-2 (SGLT2) inhibitors (comparator) who were 18 to 84 years old during 2013 to 2021 (eTable 1 in Supplement 1). Patients entered the cohort at the time of their first filled prescription for either a GLP-1 receptor agonist or an SGLT2 inhibitor during the study period. SGLT2 inhibitors were used as the comparator because this drug has no known association with suicide death and was used in similar clinical situations (predominantly as second-line or third-line glucose-lowering drugs for treatment of type 2 diabetes) during the study period, with both GLP-1 receptor agonists and SGLT2 inhibitors being recommended for patients with type 2 diabetes and high cardiovascular risk. Both drugs were primarily used for type 2 diabetes during the study period.

New use was defined as initiation of either drug among patients with no previous use of either drug at any time prior to cohort entry. Exclusion criteria were end-stage illness (severe malnutrition, cachexia, dementia, coma), history of dialysis, kidney transplant, major pancreatic disease, use of preparation of liraglutide with obesity indication before cohort entry (the comparator, SGLT2 inhibitors, are not used for this indication; liraglutide with obesity indication was defined using specific product codes), and no health care contact in the past year (to ensure a minimum level of health care contact and registration of health data), defined as neither outpatient care contact, hospital admission, nor use of any prescription drug (eTable 2 in Supplement 1).

### Study Outcomes

The primary outcome was suicide death (primary or contributing cause of death), including both confirmed suicides and self-inflicted deaths of undetermined intent^17,18^ (eTable 3 in Supplement 1). Secondary outcomes were a composite of suicide death and nonfatal self-harm recorded as a physician-assigned diagnosis during hospitalizations or outpatient hospital visits.^19^ Self-harm was also analyzed as a separate outcome. Although nonfatal self-harm is known to be underreported in Denmark,^19^ we used this outcome also in Denmark because underreporting was unlikely to differ between the study drugs, thus allowing for calculation of relative risk estimates. For the analyses of outcomes including self-harm, we excluded patients with self-harm within 3 months prior to cohort entry (eTable 2 in Supplement 1). This exclusion was done to avoid outcome misclassification: potential follow-up health care visits after a self-harm event may be registered with self-harm as the diagnosis and could be erroneously regarded as a new event in the analysis. Another secondary outcome was a composite of incident depression and anxiety-related disorders, recorded as diagnoses during hospitalizations or outpatient visits, or filled prescriptions for antidepressants (eTable 3 in Supplement 1). For this analysis, we further excluded patients with previous psychiatric disorders, defined as any psychiatric disease diagnosis or use of psychiatric medications at any time prior to cohort entry (eTable 4 in Supplement 1).

### Statistical Analysis

Patients were considered as exposed to the drug that they initiated at cohort entry until end of follow-up and were followed to outcome event, emigration, 5 years of follow-up, or end of study period (December 31, 2021). In each country separately, we estimated a propensity score for the probability of GLP-1 receptor agonist treatment conditional on variables at cohort entry, covering sociodemographic characteristics, calendar year of cohort entry, and medical history, including psychiatric diagnoses, recent health care contacts for psychiatric conditions, and prescription drug use (eTable 5 in Supplement 1). We controlled for confounding using the propensity score and standardized mortality ratio weighting to estimate the average treatment effect among the treated, as this can directly inform clinical decision-making.^20^ After exclusion of patients outside of the common range of the propensity score for the 2 groups, and those at the 1% tails of the distribution of the common range (trimming),^21,22^ the propensity score was reestimated, and those outside of the common range of the new propensity score were excluded. Reestimation of the propensity score after trimming is important because the model derived from the untrimmed population is mis-specified in the population that remains after trimming.^21^ Standardized differences below 10% after weighting were considered as good balance between exposure groups. We estimated hazard ratios (HRs) using Cox regression, with days since cohort entry as the time scale. We performed a meta-analysis of the country-specific estimates, with a fixed-effect model using the method of Mantel and Haenszel. The absolute risk difference for the primary outcome was calculated as HR − 1 multiplied by the rate in the comparator group.

We performed a subgroup analysis for the primary outcome by history of psychiatric disorders (eTable 4 in Supplement 1), as the investigated safety signal is of most importance for treatment decisions among those at high risk.^23^ We also performed subgroup analyses for those who initiated liraglutide and semaglutide, respectively. Propensity scores were reestimated for each secondary outcome and subgroup analysis.

We performed additional analyses for the primary and secondary outcomes. We applied an as-treated exposure definition, in which patients were censored at switch-to or add-on treatment with the other study drug or treatment discontinuation. Treatment duration was estimated based on the number of days covered by the filled prescriptions plus a 90-day period between prescriptions and after the last prescription. Furthermore, we restricted the follow-up to 1 year to assess whether a possible risk increase emerges shortly after treatment initiation.

Due to remaining imbalance in 1 baseline variable after weighting in Denmark (no use of other glucose-lowering drugs in the past 6 months), we adjusted for this variable in Denmark in post hoc sensitivity analysis of the primary and secondary outcomes. As the secondary outcome of incident depression and anxiety-related disorders included use of antidepressants, which can be prescribed also for other conditions, we performed a sensitivity analysis defining the outcome using only diagnoses registered during health care visits (eTable 3 in Supplement 1).

As the primary outcome analysis showed no statistically significant association, we calculated the E-value representing the minimum strength of association that an unmeasured confounder would need to have with both GLP-1 receptor agonist treatment and suicide death (conditional on the covariates included in the propensity score) to shift the confidence interval toward an increased risk such that it excludes the null.^24^ Confidence intervals not including 1.0 were considered as showing a statistically significant risk difference. Data were analyzed from March to June 2024, and analyses were conducted using SAS, version 9.4 (SAS Institute).

## Results

### Population Characteristics

**Figure 1.**
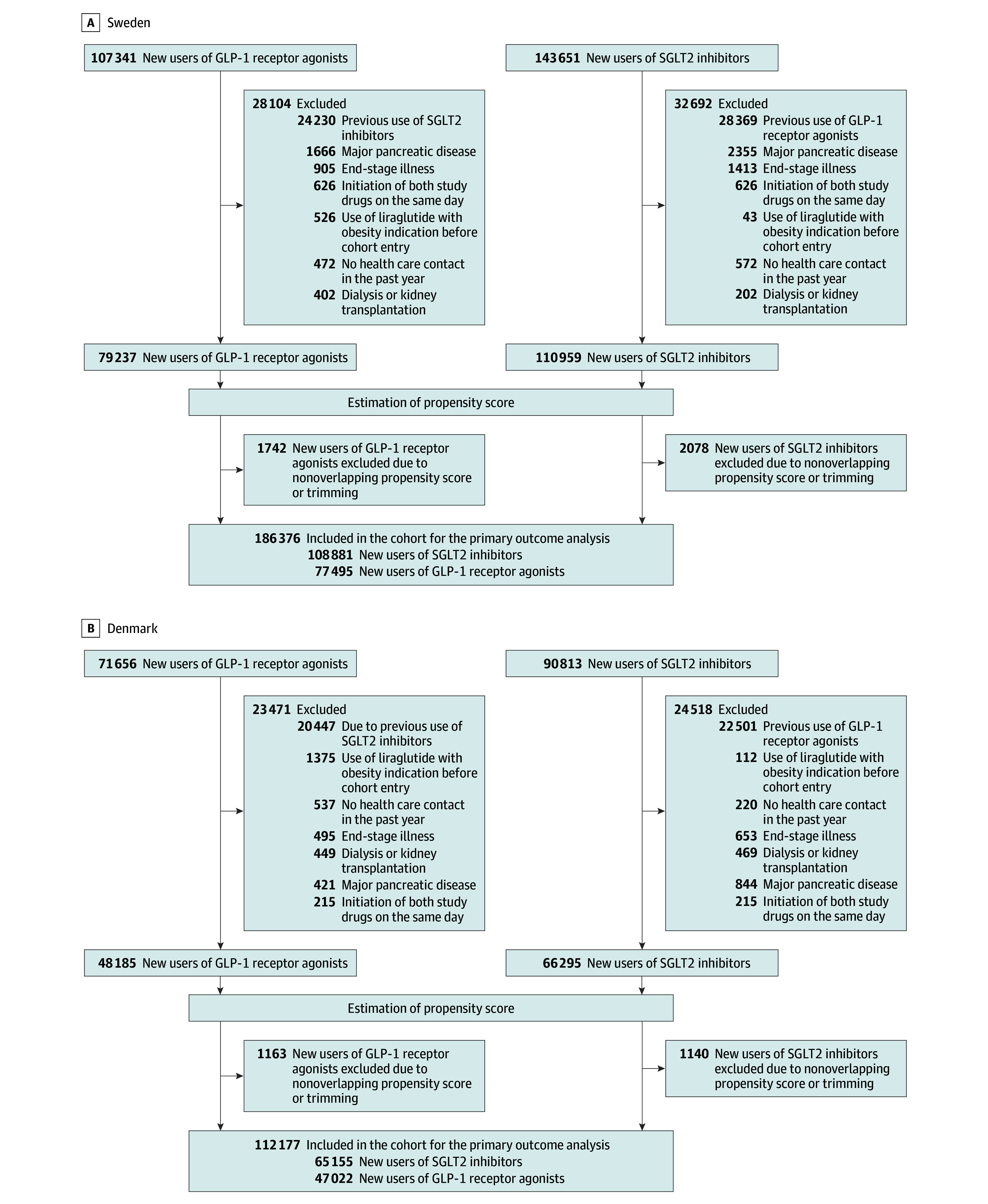
Flowchart of the Study Cohort for the Primary Outcome Analysis GLP-1 indicates glucagon-like peptide-1; SGLT2, sodium-glucose cotransporter-2.

In total, 124 517 users of GLP-1 receptor agonists and 174 036 users of SGLT2 inhibitors were included (Figure 1). Among GLP-1 receptor agonist users, the mean (SD) age was 60 (13) years and 45% were women. The most commonly used GLP-1 receptor agonists were liraglutide (50%) and semaglutide (41%). Patient characteristics before and after weighting for Sweden and Denmark separately are summarized in Table 1. The propensity score distributions are shown in eFigures 1 and 2 in Supplement 1. Patient characteristics were well balanced after weighting, except for the proportion of patients in Denmark with no use of other glucose-lowering drugs in the past 6 months (Table 1). The median (IQR) follow-up time in the primary outcome analysis was 2.8 (1.2-4.8) years for GLP-1 receptor agonist users and 2.1 (0.8-3.6) years for SGLT2 inhibitor users in Sweden, and 2.1 (0.8-4.7) years for GLP-1 receptor agonist users and 2.2 (0.8-3.9) years for SGLT2 inhibitor users in Denmark. Combining the 2 countries, mean (SD) follow-up was 2.5 (1.7) years: 2.7 (2.3) years for GLP-1 receptor agonists and 2.3 (1.6) years for SGLT2 inhibitors.

**Table 1.** Population Characteristics Before and After Propensity Score Weighting

Characteristic	No. (%)									
	Sweden					Denmark				
	GLP-1 receptor agonists (n = 77 495)	Before weighting		After weighting^a^		GLP-1 receptor agonists (n = 47 022)	Before weighting		After weighting^a^	
		SGLT2 inhibitors (n = 108 881)	Standardized difference	SGLT2 inhibitors, %	Standardized difference		SGLT2 inhibitors (n = 65 155)	Standardized difference	SGLT2 inhibitors, %	Standardized difference
Age, mean (SD), y	60.3 (12.6)	64.5 (11.1)	35.4	60.2 (10.5)	0.9	58.4 (13.1)	62.7 (11.7)	34.6	58.8 (10.8)	2.9
Sex										
Female	33 467 (43.2)	37 060 (34.0)	18.9	43.4	0.5	22 248 (47.3)	23 562 (36.2)	22.8	46.2	2.2
Male	44 028 (56.8)	71 821 (66.0)	18.9	56.6	0.5	24 774 (52.7)	41 593 (63.8)	22.8	53.8	2.2
Place of birth										
Scandinavia	62 495 (80.6)	84 642 (77.7)	7.2	80.9	0.7	41 600 (88.5)	54 671 (83.9)	13.2	88.5	0.1
Rest of Europe	5634 (7.3)	9151 (8.4)	4.2	7.2	0.3	2501 (5.3)	4541 (7.0)	6.9	5.4	0.2
Outside of Europe	9366 (12.1)	15 088 (13.9)	5.3	11.9	0.6	2921 (6.2)	5943 (9.1)	11.0	6.2	0.2
Living with partner										
Yes	37 636 (48.6)	56 679 (52.1)	7.0	48.2	0.7	29 466 (62.7)	41 118 (63.1)	0.9	62.4	0.5
No	39 859 (51.4)	52 202 (47.9)	7.0	51.8	0.7	17 556 (37.3)	24 037 (36.9)	0.9	37.6	0.5
Education										
Primary school/high school	57 847 (74.6)	82 591 (75.9)	2.8	75.1	1.0	35 902 (76.4)	51 425 (78.9)	6.2	77.2	1.9
Vocational or short tertiary education	8598 (11.1)	11 576 (10.6)	1.5	10.9	0.5	1695 (3.6)	2230 (3.4)	1.0	3.5	0.6
Medium or long tertiary education	9724 (12.5)	12 400 (11.4)	3.6	12.2	1.0	8028 (17.1)	9362 (14.4)	7.4	16.3	2.2
Missing	1326 (1.7)	2314 (2.1)	3.0	1.8	0.5	1397 (3.0)	2138 (3.3)	1.8	3.1	0.6
Cohort entry, y										
2013-2015	12 979 (16.7)	6269 (5.8)	35.3	17.7	2.5	8600 (18.3)	5132 (7.9)	31.3	19.6	3.4
2016-2018	24 699 (31.9)	32 500 (29.8)	4.4	32.2	0.8	10 221 (21.7)	20 101 (30.9)	20.8	22.5	1.8
2019-2021	39 817 (51.4)	70 112 (64.4)	26.6	50.1	2.6	28 201 (60.0)	39 922 (61.3)	2.7	57.9	4.3
History of psychiatric conditions or use of antidepressants										
Current use of antidepressants (past 6 mo)	14 705 (19.0)	15 992 (14.7)	11.5	19.5	1.2	7665 (16.3)	8196 (12.6)	10.6	16.4	0.3
Previous use of antidepressants (past 10 y but not within 6 mo)	25 760 (33.2)	29 556 (27.1)	13.3	33.9	1.4	14 711 (31.3)	16 446 (25.2)	13.5	31.5	0.5
Depression or anxiety-related disorder within past y	1975 (2.5)	1673 (1.5)	7.2	2.6	0.6	367 (0.8)	389 (0.6)	2.2	0.7	0.4
Previous depression or anxiety-related disorder (at any time before cohort entry, including past y)	9947 (12.8)	9896 (9.1)	12.0	13.2	1.1	2246 (4.8)	2240 (3.4)	6.7	4.8	0.1
Drug misuse within past y	295 (0.4)	276 (0.3)	2.3	0.4	0.4	242 (0.5)	207 (0.3)	3.1	0.6	1.1
Previous drug misuse (at any time before cohort entry, including past y)	1346 (1.7)	1272 (1.2)	4.8	1.9	1.1	699 (1.5)	657 (1.0)	4.3	1.7	1.4
Alcohol-related disorders within past y	629 (0.8)	707 (0.6)	1.9	0.9	0.7	380 (0.8)	437 (0.7)	1.6	0.8	0.4
Previous alcohol-related disorders (at any time before cohort entry, including past y)	2922 (3.8)	3578 (3.3)	2.6	3.9	0.4	1650 (3.5)	2208 (3.4)	0.7	3.5	0.1
Previous self-harm	1591 (2.1)	1548 (1.4)	4.8	2.1	0.5	79 (0.2)	91 (0.1)	0.7	0.1	0.5
Schizophrenia	1519 (2.0)	1673 (1.5)	3.2	2.1	0.7	611 (1.3)	598 (0.9)	3.6	1.2	0.5
Behavioral syndromes associated with physiological disturbances and physical factors	1067 (1.4)	919 (0.8)	5.1	1.3	0.4	176 (0.4)	162 (0.2)	2.3	0.3	0.5
Disorders of adult personality and behavior	1155 (1.5)	891 (0.8)	6.3	1.6	1.0	272 (0.6)	164 (0.3)	5.1	0.6	0.0
Other psychiatric diagnoses	2158 (2.8)	1788 (1.6)	7.8	3.0	1.0	358 (0.8)	260 (0.4)	4.8	0.8	0.3
Outpatient visit for psychiatric diagnosis during past y	4301 (5.6)	3877 (3.6)	9.6	5.8	1.0	811 (1.7)	754 (1.2)	4.8	1.7	0.6
Hospitalization for psychiatric diagnosis during past y	755 (1.0)	754 (0.7)	3.1	1.0	0.2	441 (0.9)	448 (0.7)	2.8	0.9	0.6
Outpatient visit for psychiatric diagnosis (but not during past y)	10 546 (13.6)	10 376 (9.5)	12.8	14.0	1.2	1502 (3.2)	1505 (2.3)	5.4	3.1	0.7
Hospitalization for psychiatric diagnosis (but not during past y)	3655 (4.7)	3748 (3.4)	6.4	4.9	0.8	926 (2.0)	981 (1.5)	3.5	1.9	0.2
Other medical history										
Cardiovascular disease	22 271 (28.7)	44 685 (41.0)	26.0	27.9	1.8	13 484 (28.7)	23 869 (36.6)	17.0	29.4	1.7
Diabetes complications	28 500 (36.8)	37 921 (34.8)	4.1	37.3	1.1	14 609 (31.1)	19 577 (30.0)	2.2	33.5	5.3
Obesity diagnosis	14 600 (18.8)	10 726 (9.9)	25.9	19.1	0.8	10 813 (23.0)	8169 (12.5)	27.6	23.4	0.9
Thyroid disease	1456 (1.9)	1524 (1.4)	3.8	1.9	0.2	1134 (2.4)	1238 (1.9)	3.5	2.4	0.2
Prescription drug use in past y										
β-Blocker	30 293 (39.1)	50 425 (46.3)	14.6	38.7	0.9	11 649 (24.8)	20 505 (31.5)	14.9	25.6	1.9
Opiate	13 994 (18.1)	15 743 (14.5)	9.8	18.6	1.3	8302 (17.7)	9470 (14.5)	8.5	18.2	1.4
Antipsychotic	3017 (3.9)	2979 (2.7)	6.5	4.1	1.0	2473 (5.3)	2751 (4.2)	4.9	5.4	0.5
Anxiolytic	7287 (9.4)	8462 (7.8)	5.8	9.6	0.7	1966 (4.2)	2348 (3.6)	3.0	4.3	0.7
Hypnotic or sedative	12 688 (16.4)	15 578 (14.3)	5.7	16.5	0.4	3505 (7.5)	4165 (6.4)	4.2	7.4	0.3
ADHD medications	702 (0.9)	460 (0.4)	6.0	0.9	0.4	402 (0.9)	284 (0.4)	5.2	0.8	0.5
Oral glucocorticoid	7348 (9.5)	9525 (8.7)	2.5	9.6	0.3	2974 (6.3)	3893 (6.0)	1.5	6.4	0.5
Diabetes drugs in past 6 mo										
No diabetes drug	8903 (11.5)	14 334 (13.2)	5.1	9.8	5.6	9689 (20.6)	8358 (12.8)	21.0	16.4	11.0
DPP4 inhibitors	19 006 (24.5)	27 261 (25.0)	1.2	26.1	3.6	11 453 (24.4)	19 578 (30.0)	12.8	26.8	5.6
Metformin	53 842 (69.5)	81 458 (74.8)	11.9	69.8	0.6	33 059 (70.3)	52 873 (81.1)	25.5	73.2	6.4
Sulfonylureas	9527 (12.3)	14 793 (13.6)	3.9	12.5	0.6	5627 (12.0)	8322 (12.8)	2.4	12.7	2.4
Insulin	30 449 (39.3)	23 550 (21.6)	39.1	41.4	4.2	9458 (20.1)	6585 (10.1)	28.2	22.2	5.2
Other antidiabetics	3345 (4.3)	4935 (4.5)	1.1	4.4	0.4	150 (0.3)	210 (0.3)	0.1	0.3	0.5
Health care utilization in past y										
No. of drugs used										
1-5	14 481 (18.7)	22 856 (21.0)	5.8	17.9	1.9	13 308 (28.3)	17 816 (27.3)	2.1	26.1	4.9
6-10	27 908 (36.0)	43 534 (40.0)	8.2	35.9	0.2	18 807 (40.0)	28 446 (43.7)	7.4	40.5	1.0
11-15	20 075 (25.9)	26 246 (24.1)	4.2	26.0	0.3	9924 (21.1)	13 386 (20.5)	1.4	22.0	2.1
≥16	15 031 (19.4)	16 245 (14.9)	11.9	20.1	1.8	4983 (10.6)	5507 (8.5)	7.3	11.4	2.7
No. of outpatient physician visits										
0	28 621 (36.9)	43 527 (40.0)	6.3	37.2	0.5	12 215 (26.0)	19 944 (30.6)	10.3	25.2	1.8
1-3	31 184 (40.2)	43 172 (39.7)	1.2	40.2	0.1	16 478 (35.0)	22 283 (34.2)	1.8	35.1	0.2
≥4	17 690 (22.8)	22 182 (20.4)	6.0	22.6	0.5	18 329 (39.0)	22 928 (35.2)	7.9	39.7	1.5
No. of hospital admissions										
0	63 628 (82.1)	86 279 (79.2)	7.3	82.5	1.0	33 210 (70.6)	45 301 (69.5)	2.4	70.6	0.2
1-2	11 533 (14.9)	18 265 (16.8)	5.2	14.5	1.0	10 185 (21.7)	13 613 (20.9)	1.9	21.8	0.3
≥3	2334 (3.0)	4337 (4.0)	5.3	3.0	0.2	3627 (7.7)	6241 (9.6)	6.6	7.7	0.2
Hospital admission ≤30 d before cohort entry	2612 (3.4)	6337 (5.8)	11.7	3.1	1.7	2805 (6.0)	5178 (7.9)	7.8	5.9	0.1

Abbreviations: ADHD, attention–deficit/hyperactivity disorder; DPP4, dipeptidyl peptidase 4; GLP-1, glucagon-like peptide-1; SGLT2, sodium-glucose cotransporter-2.

^a^ Determined by standardized mortality ratio weighting in which weights were set to 1 for the GLP-1 receptor agonist users (no weighting), whereas the comparator group of SGLT2 inhibitor users was weighted according to propensity score to resemble GLP-1 users.

### Primary Outcome Analysis

**Figure 2.**
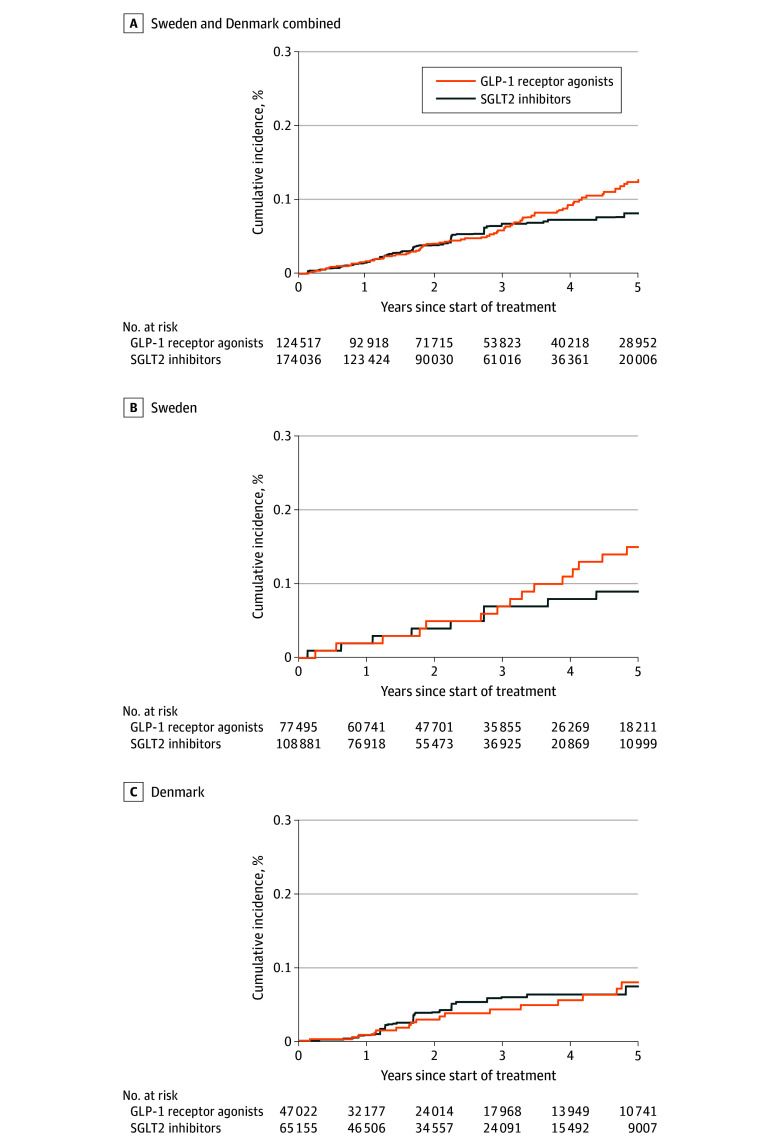
Weighted Cumulative Incidence for Suicide Death Among Users of Glucagon-Like Peptide-1 (GLP-1) Receptor Agonists and Sodium-Glucose Cotransporter-2 (SGLT2) Inhibitors in Sweden and Denmark Standardized mortality ratio weighting using a propensity score.

During follow-up, 77 GLP-1 receptor agonist users and 71 SGLT2 inhibitor users died by suicide. The weighted incidence rate was 0.23 vs 0.18 events per 1000 person-years (HR, 1.25; 95% CI, 0.83-1.88), with an absolute difference of 0.05 (95% CI, −0.03 to 0.16) events per 1000 person-years (Table 2 and Figure 2). The country-specific HR was 1.44 (95% CI, 0.87-2.37) for Sweden and 0.94 (95% CI, 0.46-1.91) for Denmark. In the subgroup analysis, the HR was 1.25 (95% CI, 0.77-2.02) for those with a history of psychiatric disorders and 1.44 (95% CI, 0.71-2.92) for those without such a history. The HR was 1.35 (95% CI, 0.85-2.15) for those initiating liraglutide and 0.74 (95% CI, 0.33-1.67) for those initiating semaglutide (Table 2).

**Table 2.** Association of Glucagon-Like Peptide-1 (GLP-1) Receptor Agonist Use vs Sodium-Glucose Cotransporter-2 (SGLT2) Inhibitor Use With the Primary Outcome of Suicide Death

Primary outcome	GLP-1 receptor agonists			SGLT2 inhibitors			Weighted hazard ratio (95% CI)^a^	Weighted absolute incidence rate difference per 1000 person-years (95% CI)^a^
	No. of users	Events, No. (%)	Weighted incidence rate per 1000 person-years^a^	No. of users	Events, No. (%)	Weighted incidence rate per 1000 person-years^a^		
Main analysis	124 517	77 (0.06)	0.23	174 036	71 (0.04)	0.18	1.25 (0.83 to 1.88)	0.05 (−0.03 to 0.16)
Analyses by country								
Sweden	77 495	60 (0.08)	0.28	108 881	46 (0.04)	0.19	1.44 (0.87 to 2.37)	0.08 (−0.02 to 0.26)
Denmark	47 022	17 (0.04)	0.15	65 155	25 (0.04)	0.16	0.94 (0.46 to 1.91)	−0.01 (−0.09 to 0.15)
Subgroup analyses								
History of psychiatric disorder	52 090	58 (0.11)	0.43	62 843	51 (0.08)	0.33	1.25 (0.77 to 2.02)	0.08 (−0.08 to 0.34)
No history of psychiatric disorder	72 420	18 (0.02)	0.09	111 083	20 (0.02)	0.07	1.44 (0.71 to 2.92)	0.03 (−0.02 to 0.13)
Initiating liraglutide	62 725	53 (0.08)	0.23	65 875	40 (0.06)	0.16	1.35 (0.85 to 2.15)	0.06 (−0.02 to 0.19)
Initiating semaglutide	49 478	8 (0.02)	0.14	49 176	13 (0.03)	0.19	0.74 (0.33 to 1.67)	−0.05 (−0.13 to 0.13)
Additional analyses								
As-treated exposure definition	124 517	41 (0.03)	0.19	174 036	37 (0.02)	0.15	1.27 (0.76 to 2.15)	0.04 (−0.04 to 0.17)
Analysis restricted to first y of follow-up	124 517	18 (0.01)	0.17	174 036	23 (0.01)	0.15	1.11 (0.54 to 2.28)	0.02 (−0.07 to 0.20)

^a^ Standardized mortality ratio weighting using a propensity score.

### Secondary Outcome Analyses

After exclusion of those with nonfatal self-harm within 3 months prior to cohort entry, 124 459 users of GLP-1 receptor agonists and 173 985 users of SGLT2 inhibitors were included in the analysis of self-harm (patient characteristics are summarized in eTable 6 in Supplement 1). The HR was 0.83 (95% CI, 0.70-0.97) for the composite outcome of suicide death and nonfatal self-harm and 0.77 (95% CI, 0.65-0.91) for self-harm (Table 3 and eFigures 3 and 4 in Supplement 1).

**Table 3.** Association of Glucagon-Like Peptide-1 (GLP-1) Receptor Agonist Use vs Sodium-Glucose Cotransporter-2 (SGLT2) Inhibitor Use With Secondary Outcomes

Secondary outcome	GLP-1 receptor agonists			SGLT2 inhibitors			Weighted hazard ratio (95% CI)^a^
	No. of users	Events, No. (%)	Weighted incidence rate per 1000 person-years^a^	No. of users	Events, No. (%)	Weighted incidence rate per 1000 person-years^a^	
**Suicide death and nonfatal self-harm**							
Main analysis	124 459	489 (0.39)	1.47	173 985	465 (0.27)	1.78	0.83 (0.70-0.97)
Additional analyses							
As-treated exposure definition	124 459	276 (0.22)	1.28	173 985	259 (0.15)	1.54	0.85 (0.69-1.05)
Analysis restricted to first year of follow-up	124 459	157 (0.13)	1.46	173 985	193 (0.11)	2.22	0.66 (0.51-0.86)
**Self-harm**							
Main analysis	124 459	419 (0.34)	1.26	173 985	404 (0.23)	1.62	0.77 (0.65-0.91)
Additional analyses							
As-treated exposure definition	124 459	238 (0.19)	1.10	125 668	286 (0.23)	1.42	0.80 (0.64-1.01)
Analysis restricted to first year of follow-up	124 459	141 (0.11)	1.31	125 668	232 (0.18)	2.11	0.63 (0.48-0.83)
**Incident depression and anxiety-related disorders**							
Main analysis	72 420	4913 (6.78)	25.8	111 083	5848 (5.26)	25.4	1.01 (0.97-1.06)
Additional analyses							
As-treated exposure definition	72 420	2944 (4.07)	22.9	111 083	3393 (3.05)	22.2	1.04 (0.98-1.10)
Analysis restricted to first year of follow-up	72 420	1619 (2.24)	25.9	111 083	2061 (1.86)	24.3	1.07 (0.98-1.16)
Sensitivity analysis							
Outcome definition restricted to diagnoses registered during health care visits	72 420	501 (0.69)	2.53	111 083	442 (0.40)	2.14	1.17 (1.00-1.37)

^a^ Standardized mortality ratio weighting using a propensity score.

After exclusion of those with previous psychiatric disorders, 72 420 users of GLP-1 receptor agonists and 111 083 users of SGLT2 inhibitors were included in the analysis of incident depression and anxiety-related disorders (patient characteristics are summarized in eTable 7 in Supplement 1). The HR was 1.01 (95% CI, 0.97-1.06) (Table 3 and eFigure 5 in Supplement 1). Results for Sweden and Denmark separately are summarized in eTable 8 (primary outcome) and eTable 9 (secondary outcomes) in Supplement 1.

### Additional Analyses and Sensitivity Analysis

In the analyses of suicide death, the HR was 1.27 (95% CI, 0.76-2.15) when using the as-treated exposure definition (mean [SD] follow-up time was 1.7 [1.5] years for GLP-1 receptor agonists and 1.5 [1.3] years for SGLT2 inhibitors) and 1.11 (95% CI, 0.54-2.28) when restricting the follow-up to the first year (Table 2). The post hoc sensitivity analysis, in which the variable no use of other glucose-lowering drug in the past 6 months was adjusted for, yielded an HR consistent with that of the main analysis in Denmark (eTable 8 in Supplement 1). The additional analyses for the secondary outcomes and the sensitivity analysis in which the secondary outcome of incident depression and anxiety-related disorders was restricted to diagnoses registered during health care visits are summarized in Table 3. The E-value for the primary analysis showed that an unmeasured confounder would need to be associated with both GLP-1 receptor agonist use and suicide death by a risk ratio of at least 1.7 to shift the confidence interval to exclude the null.

## Discussion

In this cohort study of nationwide data from 2 countries, we found no statistically significant increased risk of suicide death for GLP-1 receptor agonists vs SGLT2 inhibitors used predominantly for type 2 diabetes. The upper limit of the confidence interval was compatible with up to an 88% relative-risk increase of suicide death, which corresponded to an absolute risk increase of no more than 0.16 per 1000 person-years. The findings indicate that the absolute risks of suicide death in broad groups of patients using GLP-1 receptor agonists are low and any potential risk increase would be small. We also found a slightly lower risk of self-harm and no statistically significant association with incident depression and anxiety-related disorders.

The investigated safety concern for GLP-1 receptor agonist use was based on spontaneous reports of suicidal thoughts and thoughts of self-harm.^1^ To cover a wider range of conditions and outcomes associated with thoughts of self-harm and suicide,^25,26^ we assessed suicide death, suicide death and nonfatal self-harm, and self-harm and incident depression and anxiety-related disorders. In randomized clinical trials of GLP-1 receptor agonists, increases in suicidality, depression, anxiety, and other adverse mental health outcomes have not been observed.^27,28^ However, many trials excluded those at highest risk of suicidality through exclusion criteria or based on investigators’ judgment such that uncertainty remained regarding the generalizability of the findings to broader patient groups, and the statistical power was low due to few events. For example, a meta-analysis of clinical trials of GLP-1 receptor agonists included only 18 events of suicidal behavior among exposed patients.^28^

A previous study^29^ using electronic health records from the TriNetX Analytics Network assessed diagnoses of suicidal ideation during 6 months after treatment initiation with the GLP-1 receptor agonist semaglutide. In separate analyses, the study included 52 783 patients with overweight or obesity and 27 282 patients with type 2 diabetes who were prescribed semaglutide, then propensity score matched them with users of non–GLP-1 receptor agonist antiobesity or glucose-lowering medications. Semaglutide use was associated with very large relative reductions in suicidal ideation diagnosis of around 70% in both sets of analyses, indicating that the study design likely introduced important biases. For example, immortal time bias^30^ may have been introduced as a new-user design was not used: patients with semaglutide prescriptions were first included regardless of their comparator drug use history, and the comparator group was selected from the remaining patients prescribed the comparator drug (ie, the exposure status for the comparator group was conditioned on not being prescribed semaglutide after cohort entry). Moreover, in the analyses of patients with type 2 diabetes, any other glucose-lowering drugs, including those used as first-line therapies, were included as the comparator, although this may have led to time-lag bias through misalignment in disease progression between the groups.^31^ Furthermore, suicide death during follow-up could not be accounted for as data on vital status and causes of death were not available.

### Strengths and Limitations

This study expands the knowledge regarding the safety of GLP-1 receptor agonists by providing data about the risk of suicide death associated with use of the drugs using an active-comparator new-user design,^16^ which avoids time-related biases^31^ and aligns patients in the exposed vs control group with respect to disease stage. Moreover, confounders were adjusted for using a propensity score, including a wide range of patient characteristics, and the nationwide registers enabled analysis of national study populations with virtually complete data on cause of death.^13,14^ This study supports the conclusions of the European Medicines Agency’s investigation and the US Food and Drug Administration’s preliminary evaluation^32^ that the available evidence does not support a causal association between use of GLP-1 receptor agonists and suicidal and self-injurious thoughts and actions.^33^

This study has limitations. We adjusted for potential confounders, including psychiatric disorders and socioeconomic status recorded in the registers, but unmeasured confounding may have affected the results. During the study period, GLP-1 receptor agonists were predominantly used for treatment of type 2 diabetes. The drugs are increasingly used also in patients with obesity who do not have diabetes, and the study findings might not be generalizable to this group of patients. Although it is possible that some patients included in the study used GLP-1 receptor agonists off label for weight reduction, this proportion is likely to be low; a Danish study showed that the proportion of new users of semaglutide with no evidence of a type 2 diabetes diagnosis in the register data (a glycated hemoglobin value in the diabetic range, previous use of glucose-lowering medications, or diagnosis during hospital contacts) was 1% to 5% in 2018 to 2020 and 15% in 2021.^34^ Moreover, in the present analyses, liraglutide (50%) and semaglutide (41%) were the most used GLP-1 receptor agonists; the subgroup analyses for each of the 2 GLP-1 receptor agonists had low numbers of events, and associations with suicidality could differ between individual drugs. Furthermore, mean follow-up time for GLP-1 receptor agonist users was 2.7 years, and although 25% of the GLP-1 receptor agonist users were followed for at least 4.7 years, risks may emerge with longer-term use. Although subgroup analyses with low numbers of events should be interpreted with much caution, especially when the subgroup hypothesis was not formulated a priori based on a suggested mechanism,^35^ the HR for suicide death in Sweden was 1.44 (95% CI, 0.87-2.37), with this nominal risk difference emerging around 3 years after cohort entry.

Some suicide deaths could also be misclassified.^36^ In an assessment of the reliability of the cause of death register from 2008, the proportion of suicide deaths with intent confirmed by experts was 81% in Sweden and 90% in Denmark, with few accidents and natural deaths being reclassified as suicides.^36^ We also included self-inflicted deaths of undetermined intent in the primary outcome definition, as an investigation by the National Center for Suicide Research and Prevention in Sweden showed that 20% of suicide cases in the country were coded as of undetermined intent and 70% to 75% of self-inflicted deaths with unknown intent were reclassified as suicide after further investigation.^18^ The overall rates of suicide death in the study population were largely in line with national estimates in both Denmark^37^ and Sweden.^38^ Nonfatal self-harm is known to be underreported in Denmark,^19^ and the absolute risks of the secondary outcome analysis, including this outcome, are likely underestimated, as is demonstrated by the substantially lower rates observed in Denmark compared to Sweden. We could also not assess suicidal thoughts and self-harm that did not result in suicide death or a registered diagnosis during contact with the health care system. The low risk of suicide death meant that the study had limited power to assess smaller risk increases.

## Conclusions

In this binational cohort study including predominantly patients with type 2 diabetes, use of GLP-1 receptor agonists compared with SGLT2 inhibitors was not associated with an increased risk of suicide death, self-harm, or incident depression and anxiety-related disorders. While reassuring, the study could not rule out smaller absolute risk differences for suicide death, and future studies with more outcome events should be performed as data accumulate.

## Notes

Supplement 1. **eTable 1.** ATC codes and estimated days of exposure per unit of GLP-1 receptor agonists and SGLT2 inhibitors **eTable 2.** ICD-10 and procedure codes for exclusion criteria. **eTable 3.** Outcome definitions **eTable 4.** Definition of psychiatric disorders for subgroup analysis of the primary outcome analysis and exclusion criteria for secondary outcome analysis of incident depression and anxiety-related disorder **eTable 5.** Propensity score variables and definitions **eTable 6.** Population characteristics for secondary outcome analyses of suicide death and non-fatal self-harm, and self-harm **eTable 7.** Population characteristics for secondary outcome analysis of incident depression and anxiety-related disorders **eTable 8.** Main, additional and sensitivity analyses of the primary outcome of suicide death in Sweden and Denmark **eTable 9.** Secondary outcome analyses in Sweden and Denmark **eFigure 1.** Propensity score distribution in the primary outcome analysis in Sweden **eFigure 2.** Propensity score distribution in the primary outcome analysis in Denmark **eFigure 3.** Weighted cumulative incidence for the composite of suicide death and non-fatal self-harm in Sweden and Denmark **eFigure 4.** Weighted cumulative incidence for self-harm in Sweden and Denmark **eFigure 5.** Weighted cumulative incidence for the composite of incident depression and anxiety-related disorders in Sweden and Denmark

Supplement 2. Data Sharing Statement

## References

[1] EMA statement on ongoing review of GLP-1 receptor agonists. European Medicines Agency. July 11, 2023. Accessed November 27, 2023. https://www.ema.europa.eu/en/news/ema-statement-ongoing-review-glp-1-receptor-agonists

[2] Trapp S, Brierley DI. Brain GLP-1 and the regulation of food intake: GLP-1 action in the brain and its implications for GLP-1 receptor agonists in obesity treatment. Br J Pharmacol. 2022;179(4):557-570. doi:10.1111/bph.15638 34323288 PMC8820179

[3] Dong M, Wen S, Zhou L. The relationship between the blood-brain-barrier and the central effects of glucagon-like peptide-1 receptor agonists and sodium-glucose cotransporter-2 inhibitors. Diabetes Metab Syndr Obes. 2022;15:2583-2597. doi:10.2147/DMSO.S375559 36035518 PMC9417299

[4] Castaneda D, Popov VB, Wander P, Thompson CC. Risk of suicide and self-harm is increased after bariatric surgery—a systematic review and meta-analysis. Obes Surg. 2019;29(1):322-333. doi:10.1007/s11695-018-3493-4 30343409

[5] Coulter AA, Rebello CJ, Greenway FL. Centrally acting agents for obesity: past, present, and future. Drugs. 2018;78(11):1113-1132. doi:10.1007/s40265-018-0946-y 30014268 PMC6095132

[6] Detka J, Głombik K. Insights into a possible role of glucagon-like peptide-1 receptor agonists in the treatment of depression. Pharmacol Rep. 2021;73(4):1020-1032. doi:10.1007/s43440-021-00274-8 34003475 PMC8413152

[7] Ludvigsson JF, Almqvist C, Bonamy AKE, . Registers of the Swedish total population and their use in medical research. Eur J Epidemiol. 2016;31(2):125-136. doi:10.1007/s10654-016-0117-y 26769609

[8] Schmidt M, Pedersen L, Sørensen HT. The Danish Civil Registration System as a tool in epidemiology. Eur J Epidemiol. 2014;29(8):541-549. doi:10.1007/s10654-014-9930-3 24965263

[9] Wettermark B, Hammar N, Fored CM, . The new Swedish Prescribed Drug Register—opportunities for pharmacoepidemiological research and experience from the first six months. Pharmacoepidemiol Drug Saf. 2007;16(7):726-735. doi:10.1002/pds.1294 16897791

[10] Pottegård A, Schmidt SAJ, Wallach-Kildemoes H, Sørensen HT, Hallas J, Schmidt M. Data resource profile: the Danish National Prescription Registry. Int J Epidemiol. 2017;46(3):798-798f. 27789670 10.1093/ije/dyw213 PMC5837522

[11] Ludvigsson JF, Andersson E, Ekbom A, . External review and validation of the Swedish national inpatient register. BMC Public Health. 2011;11(1):450. doi:10.1186/1471-2458-11-450 21658213 PMC3142234

[12] Schmidt M, Schmidt SAJ, Sandegaard JL, Ehrenstein V, Pedersen L, Sørensen HT. The Danish National Patient Registry: a review of content, data quality, and research potential. Clin Epidemiol. 2015;7:449-490. doi:10.2147/CLEP.S91125 26604824 PMC4655913

[13] Brooke HL, Talbäck M, Hörnblad J, . The Swedish cause of death register. Eur J Epidemiol. 2017;32(9):765-773. doi:10.1007/s10654-017-0316-1 28983736 PMC5662659

[14] Helweg-Larsen K. The Danish register of causes of death. Scand J Public Health. 2011;39(7)(suppl):26-29. doi:10.1177/1403494811399958 21775346

[15] Maret-Ouda J, Tao W, Wahlin K, Lagergren J. Nordic registry-based cohort studies: possibilities and pitfalls when combining Nordic registry data. Scand J Public Health. 2017;45(suppl 17):14-19. doi:10.1177/1403494817702336 28683665

[16] Lund JL, Richardson DB, Stürmer T. The active comparator, new user study design in pharmacoepidemiology: historical foundations and contemporary application. Curr Epidemiol Rep. 2015;2(4):221-228. doi:10.1007/s40471-015-0053-5 26954351 PMC4778958

[17] Neovius M, Bruze G, Jacobson P, . Risk of suicide and non-fatal self-harm after bariatric surgery: results from two matched cohort studies. Lancet Diabetes Endocrinol. 2018;6(3):197-207. doi:10.1016/S2213-8587(17)30437-0 29329975 PMC5932484

[18] Interpreting suicide data. National Centre for Suicide Research and Prevention. Accessed April 27, 2024. https://ki.se/en/nasp/interpreting-suicide-data

[19] Reuter Morthorst B, Soegaard B, Nordentoft M, Erlangsen A. Incidence rates of deliberate self-harm in Denmark 1994-2011. Crisis. 2016;37(4):256-264. doi:10.1027/0227-5910/a000391 27278571 PMC5137321

[20] Desai RJ, Franklin JM. Alternative approaches for confounding adjustment in observational studies using weighting based on the propensity score: a primer for practitioners. BMJ. 2019;367:l5657. doi:10.1136/bmj.l5657 31645336

[21] Stürmer T, Webster-Clark M, Lund JL, . Propensity score weighting and trimming strategies for reducing variance and bias of treatment effect estimates: a simulation study. Am J Epidemiol. 2021;190(8):1659-1670. doi:10.1093/aje/kwab041 33615349 PMC8327194

[22] Stürmer T, Wyss R, Glynn RJ, Brookhart MA. Propensity scores for confounder adjustment when assessing the effects of medical interventions using nonexperimental study designs. J Intern Med. 2014;275(6):570-580. doi:10.1111/joim.12197 24520806 PMC4037382

[23] Ruder K. As semaglutide’s popularity soars, rare but serious adverse effects are emerging. JAMA. 2023;330(22):2140-2142. doi:10.1001/jama.2023.16620 37966850

[24] VanderWeele TJ, Ding P. Sensitivity analysis in observational research: introducing the E-value. Ann Intern Med. 2017;167(4):268-274. doi:10.7326/M16-2607 28693043

[25] Knipe D, Padmanathan P, Newton-Howes G, Chan LF, Kapur N. Suicide and self-harm. Lancet. 2022;399(10338):1903-1916. doi:10.1016/S0140-6736(22)00173-8 35512727

[26] Klonsky ED, May AM, Saffer BY. Suicide, suicide attempts, and suicidal ideation. Annu Rev Clin Psychol. 2016;12:307-330. doi:10.1146/annurev-clinpsy-021815-093204 26772209

[27] O’Neil PM, Aroda VR, Astrup A, ; Satiety and Clinical Adiposity—Liraglutide Evidence in individuals with and without diabetes (SCALE) study groups. Neuropsychiatric safety with liraglutide 3.0 mg for weight management: results from randomized controlled phase 2 and 3a trials. Diabetes Obes Metab. 2017;19(11):1529-1536. doi:10.1111/dom.12963 28386912 PMC5655710

[28] Silverii GA, Marinelli C, Mannucci E, Rotella F. Glucagon-like peptide-1 receptor agonists and mental health: a meta-analysis of randomized controlled trials. Diabetes Obes Metab. 2024;26(6):2505-2508. doi:10.1111/dom.15538 38449004

[29] Wang W, Volkow ND, Berger NA, Davis PB, Kaelber DC, Xu R. Association of semaglutide with risk of suicidal ideation in a real-world cohort. Nat Med. 2024;30(1):168-176. doi:10.1038/s41591-023-02672-2 38182782 PMC11034947

[30] Suissa S. Immortal time bias in pharmaco-epidemiology. Am J Epidemiol. 2008;167(4):492-499. doi:10.1093/aje/kwm324 18056625

[31] Suissa S, Azoulay L. Metformin and the risk of cancer: time-related biases in observational studies. Diabetes Care. 2012;35(12):2665-2673. doi:10.2337/dc12-0788 23173135 PMC3507580

[32] Update on FDA’s ongoing evaluation of reports of suicidal thoughts or actions in patients taking a certain type of medicines approved for type 2 diabetes and obesity. US Food and Drug Administration. January 11, 2024. Accessed July 29, 2024. https://www.fda.gov/drugs/drug-safety-and-availability/update-fdas-ongoing-evaluation-reports-suicidal-thoughts-or-actions-patients-taking-certain-type

[33] Meeting highlights from the Pharmacovigilance Risk Assessment Committee (PRAC) 8-11 April 2024. European Medicines Agency. April 12, 2024. Accessed May 1, 2024. https://www.ema.europa.eu/en/news/meeting-highlights-pharmacovigilance-risk-assessment-committee-prac-8-11-april-2024

[34] Mailhac A, Pedersen L, Pottegård A, . Semaglutide (Ozempic^®^) use in Denmark 2018 through 2023—user trends and off-label prescribing for weight loss. Clin Epidemiol. 2024;16:307-318. doi:10.2147/CLEP.S456170 38685990 PMC11057509

[35] Sun X, Ioannidis JPA, Agoritsas T, Alba AC, Guyatt G. How to use a subgroup analysis: users’ guide to the medical literature. JAMA. 2014;311(4):405-411. doi:10.1001/jama.2013.285063 24449319

[36] Tøllefsen IM, Helweg-Larsen K, Thiblin I, . Are suicide deaths under-reported? nationwide re-evaluations of 1800 deaths in Scandinavia. BMJ Open. 2015;5(11):e009120. doi:10.1136/bmjopen-2015-009120 26608638 PMC4663440

[37] Konieczna A, Larsen CP, Jakobsen SG, . Suicide trends in Denmark—an ecological study exploring suicide methods from 1995 to 2019. PLoS One. 2023;18(12):e0296324. doi:10.1371/journal.pone.0296324 38157350 PMC10756527

[38] Suicides in Sweden. National Centre for Suicide Research and Prevention. Accessed April 27, 2024. https://ki.se/en/nasp/statistics/suicide-in-sweden

