# Supplementary material for: GLP-1 Receptor Agonist Use and Risk of Suicide Death
Source: JAMA Intern Med. 2024 Sep 3;184(11):1301–12. doi: 10.1001/jamainternmed.2024.4369 (PMC11372654; doi:10.1001/jamainternmed.2024.4369)
Supplement: Supplement 1. — eTable 1. ATC codes and estimated days of exposure per unit of GLP-1 receptor agonists and SGLT2 inhibitors eTable 2. ICD-10 and procedure codes for exclusion criteria. eTable 3. Outcome definitions eTable 4. Definition of psychiatric disorders for subgroup analysis of the primary outcome analysis and exclusion criteria for secondary outcome analysis of incident depression and anxiety-related disorder eTable 5. Propensity score variables and definitions eTable 6. Population characteristics for secondary outcome analyses of suicide death and non-fatal self-harm, and self-harm eTable 7. Population characteristics for secondary outcome analysis of incident depression and anxiety-related disorders eTable 8. Main, additional and sensitivity analyses of the primary outcome of suicide death in Sweden and Denmark eTable 9. Secondary outcome analyses in Sweden and Denmark eFigure 1. Propensity score distribution in the primary outcome analysis in Sweden eFigure 2. Propensity score distribution in the primary outcome analysis in Denmark eFigure 3. Weighted cumulative incidence for the composite of suicide death and non-fatal self-harm in Sweden and Denmark eFigure 4. Weighted cumulative incidence for self-harm in Sweden and Denmark eFigure 5. Weighted cumulative incidence for the composite of incident depression and anxiety-related disorders in Sweden and Denmark [file jamainternmed-e244369-s001.pdf]

Supplemental Online Content

Ueda P, Söderling J, Wintzell V, et al. GLP-1 receptor agonist use and risk of suicide death. *JAMA Intern Med*. Published online September 3, 2024.
doi:10.1001/jamainternmed.2024.4369

eTable 1. ATC codes and estimated days of exposure per unit of GLP-1 receptor agonists and SGLT2 inhibitors

eTable 2. ICD-10 and procedure codes for exclusion criteria.

eTable 3. Outcome definitions

eTable 4. Definition of psychiatric disorders for subgroup analysis of the primary outcome analysis and exclusion criteria for secondary outcome analysis of incident depression and anxiety-related disorder

eTable 5. Propensity score variables and definitions

eTable 6. Population characteristics for secondary outcome analyses of suicide death and non-fatal self-harm, and self-harm

eTable 7. Population characteristics for secondary outcome analysis of incident depression and anxiety-related disorders

eTable 8. Main, additional and sensitivity analyses of the primary outcome of suicide death in Sweden and Denmark

eTable 9. Secondary outcome analyses in Sweden and Denmark

eFigure 1. Propensity score distribution in the primary outcome analysis in Sweden

eFigure 2. Propensity score distribution in the primary outcome analysis in Denmark

eFigure 3. Weighted cumulative incidence for the composite of suicide death and non-fatal self-harm in Sweden and Denmark

eFigure 4. Weighted cumulative incidence for self-harm in Sweden and Denmark

eFigure 5. Weighted cumulative incidence for the composite of incident depression and anxiety-related disorders in Sweden and Denmark

This supplemental material has been provided by the authors to give readers additional information about their work.

© 2024 Ueda P et al. *JAMA Intern Med.*

eTable 1. ATC codes and estimated days of exposure per unit of GLP-1 receptor agonists and SGLT2 inhibitors

Drug	ATC definition	Estimated days of supply Denmark	Estimated days of supply Sweden
**GLP-1 receptor agonists**	A10BJ01, A10BJ02[a], A10BJ03, A10BJ05, A10BJ06, A10AE54, A10AE56	A10BJ01 (5 or 10 microgram) = 0.5 per dose<br>A10BJ01 (2 mg) = 7.0 per dose<br>A10BJ02 = 15.0 per pen<br>A10BJ03 = 14.0 per pen<br>A10BJ05 = 7.0 per dose<br>A10BJ06 = 28.0 per pen<br>A10AE54 = 2.5 per ml<br>A10AE56 = not available	A10BJ01 (5 or 10 microgram) = 0.5 per dose<br>A10BJ01 (2 mg) = 7.0 per dose<br>A10BJ02 = 5.0 per ml<br>A10BJ03 = 1.0 per dose<br>A10BJ05 = 7.0 per dose<br>A10BJ06 = 7.0 per dose<br>A10AE54 = 2.5 per ml<br>A10AE56 = 3.0 per ml
**SGLT2 inhibitors**	A10BK01, A10BK02, A10BK03, A10BK04, A10BD15, A10BD16, A10BD19, A10BD20, A10BD21, A10BD23, A10BD24, A10BD25	All units are days per tablet<br>A10BK01 = 1.0; A10BK02 = 1.0;<br>A10BK03 = 1.0; A10BK04 = 1.0;<br>A10BD15 = 0.5; A10BD16 = 0.5;<br>A10BD19 = 1.0; A10BD20 = 0.5;<br>A10BD21 = 1.0; A10BD23 = 0.5;<br>A10BD24 = 1.0; A10BD25 = 0.5	As in Denmark.

[a] Not including saxenda: product no.131577, 395175, 164108, 439932 and 575140 in Denmark and 513490, 141823, 439932, 026334 or 471462 in Sweden.

© 2024 Ueda P et al. *JAMA Intern Med.*

eTable 2. ICD-10 and procedure codes for exclusion criteria

	**Codes (ICD-10, procedure, or ATC)**[a]	**Data source, type of diagnosis, type of hospital contact**
Initiation of both study drugs at the same day	n.a.	Prescribed drug register
End stage illness (severe malnutrition, cachexia, dementia, coma) at any time before cohort entry	ICD-10: E40-E43, F00-F03, G30, R40.2[b], R64<br>ATC: N06D	Patient register, any position, any type of hospital contact; prescribed drug register
Dialysis or renal transplantation at any time before cohort entry	ICD-10: Z49, Z94.0[b], Z99.2<br>Procedure: KAS, JFD, JFZ	Patient register, any position, any type of hospital contact
Major pancreatic disease (chronic pancreatitis [defined by pancreatic enzyme substitution prescription within last year or diagnosis at any time before cohort entry], pancreatic cancer, major pancreatic surgery at any time before cohort entry)	ICD-10: C25, K86.0, K86.1<br>Procedure: JLC, JLE<br>ATC: A09AA02 (Denmark), A09AA (Sweden)	Patient register, any position, any type of hospital contact; prescribed drug register
Liraglutide with obesity indication (Saxenda) at any time before index date	A10BJ02 with product number 131577, 395175 164108, 439932 or 575140 in Denmark and 513490, 141823, 439932, 026334 or 471462 in Sweden)	Prescribed drug register
No specialist care contact or prescription drug in last year prior to cohort entry	N.A.	Patient register, prescribed drug register
**Additional exclusion criteria for secondary outcome analysis of suicide death and non-fatal self-harm**		
Self-harm within 3 months prior to cohort entry	X60-X84<br>Y10-34	Patient register, any position, any type of hospital contact

[a] 10-year look-back unless otherwise specified.
[b] Only available in Denmark

© 2024 Ueda P et al. *JAMA Intern Med.*

eTable 3. Outcome definitions

	ICD/ATC-codes	Data source, type of diagnosis, type of hospital contact
**Primary outcome**		
Suicide death	ICD-10: X60-X84, Y10-Y34	*Cause of Death Register*<br>Underlying or contributing cause of death
**Secondary outcomes**		
Suicide death and non-fatal self-harm	ICD-10: X60-X84, Y10-34	*Cause of Death Register*<br>Underlying or contributing cause of death<br><br>*National Patient Register*<br>Any type of visit, any position
Self-harm	ICD-10: X60-X84, Y10-34	*National Patient Register*<br>Any type of visit, any position
Incident depression and anxiety-related disorders	ICD-10: F32, F33, F34, F38, F39, F40-F45, F48<br><br>ATC: N06A	*National Patient Register*<br>Any type of visit, any position<br><br>*Prescribed drug register*
Incident depression and anxiety-related disorder diagnosis (sensitivity analysis for secondary outcome)	ICD-10: F32, F33, F34, F38, F39, F40-F45, F48	*National Patient Register*<br>Any type of visit, any position

© 2024 Ueda P et al. *JAMA Intern Med.*

eTable 4. Definition of psychiatric disorders for subgroup analysis of the primary outcome analysis and exclusion criteria for secondary outcome analysis of incident depression and anxiety-related disorder

	**ICD/categories**[a]	**Data source, type of diagnosis, type of hospital contact**
Previous use of antidepressants, ADHD medication, antipsychotics, anxiolytics	ATC: N06A, N05A, N05B, N06BA (except N06BA07), C02AC02, N07BC, N07BB	Prescribed drug register, filled prescription
Diagnosis of psychiatric disorder	ICD-10: F, R78.1-R78.5[b], T40, X60-X84, Y10-34, Y870, Y872, Y899	Patient register, any position, any type of hospital contact

[a] 10-year look-back
[b] Only available in Denmark

© 2024 Ueda P et al. *JAMA Intern Med.*

eTable 5. Propensity score variables and definitions

**Sociodemographic characteristics**	**ICD-10 codes/ATC-codes/categories**	**Data source, type of diagnosis, type of hospital contact**
Sex	Women/men	
Age	Continuous	
Place of birth	Scandinavia; Rest of Europe; Outside Europe, Missing	
Living with partner	Yes/no	
Education	Primary school and high school; vocational or short-term tertiary education; medium or long tertiary education; missing	
Calendar year	3-year category	
**History of psychiatric conditions or use of antidepressants**[a] (10 yr look-back or according to specification)		
Current use of antidepressants (filled prescription within 6 months prior to cohort entry)	ATC: N06A	Prescribed drug register, filled prescription
Previous use of antidepressants (filled prescription but not within 6 months prior to cohort entry)	ATC: N06A	Prescribed drug register, filled prescription
Depression or anxiety related disorder within last year	ICD-10: F32, F33, F34, F38, F39<br>F40-F45, F48	Patient register, primary position, any type of hospital contact
Previous depression or anxiety-related disorder (at any time before cohort entry, including last year)	ICD-10: F32, F33, F34, F38, F39<br>F40-F45, F48	Patient register, any position, any type of hospital contact
Drug misuse within last year	ICD-10: F11-F16, F18, F19, R78.1-R78.5[b], T40<br>ATC: N07BC	Patient register, primary position, any type of hospital contact<br><br>Prescribed drug register, filled prescription
Previous drug misuse (at any time before cohort entry, including last year)	ICD-10: F11-F16, F18, F19, R78.1-R78.5[b], T40<br>ATC: N07BC	Patient register, any position, any type of hospital contact<br><br>Prescribed drug register, filled prescription
Alcohol related disorders within last year	ICD10: F10<br>ATC: N07BB	Patient register, primary position, any type of hospital contact<br><br>Prescribed drug register, filled prescription
Previous alcohol related disorders (at any time before cohort entry, including last year)	ICD10: F10<br><br>ATC: N07BB	Patient register, any position, any type of hospital contact<br><br>Prescribed drug register, filled prescription
Previous self-harm	X60-X84<br>Y10-34<br>Y870, Y872, Y899	Patient register, any position, any type of hospital contact

© 2024 Ueda P et al. *JAMA Intern Med.*

Schizophrenia	F20-F29	Patient register, any position, any type of hospital contact
Behavioural syndromes associated with physiological disturbances and physical factors	F50-F59	Patient register, any position, any type of hospital contact
Disorders of adult personality and behavior	F60-F69	Patient register, any position, any type of hospital contact
Other psychiatric diagnoses	F70-F99	Patient register, any position, any type of hospital contact
Outpatient visit for psychiatric diagnosis during the last year	F00-F99	Patient register, primary position, outpatient visit
Hospitalization for psychiatric diagnosis during the last year	F00-F99	Patient register, primary position, hospitalization
Outpatient visit for psychiatric diagnosis (any previous visit but not during last year)	F00-F99	Patient register, primary position, outpatient visit
Hospitalization for psychiatric diagnosis (any previous hospitalization but not during last year)	F00-F99	Patient register, primary position, hospitalization
**Other medical history** (10 yr look-back)		
Cardiovascular disease	ICD-10: I09.8, Z95.1[b], Z95.5[b], I11, I20, I21, I22, I24, I25, I130, I132, I34-I37, I42, I43, I44-I47, I48, I49, I50, I60-I69, I70, I71, I72, I73, I74, I77, J81, G45 (excl G453, G454), G46, K550[b], K551[b], E115, E145, E135<br>Procedure codes: FNA, FNB, FNC, FND, FNE, FNG, FNP02, FNP12, FNQ05, FNQ12, FNR22	Patient register, any position, any type of hospital contact
Diabetes complications	ICD-10: E110, E111, E112, E113, E114, E116, E117, E118, E130, E131, E133, E134, E136, E137, E138, E140, E141, E142, E143, E144, E146, E147, E148, E160, E161, E162, G990, G590, G632, H280, H358, H360, I120, I131, I132, L984[b], M142[b], M146[b], M908[b], N00-08, N10-N16, N17, N18, N19, N20-N23, N25-N29.<br>Procedure codes: CKC10, CKC12, CKC15, CKD65	Patient register, any position, any type of hospital contact
Obesity diagnosis	ICD-10: E66	Patient register, any position, any type of hospital contact
Thyroid disease	ICD10: E012, E018, E032, E038, E040, E041, E042, E049, E890, E06	Patient register, any position, any type of hospital contact
**Prescription drug use in the last year**		
Beta-blocker	C07	
Opiate	N02A	
Antipsychotic[a]	N05A	
Anxiolytic[a]	N05B	
Hypnotic or sedative	N05C	
ADHD medications[a]	N06BA (except N06BA07), C02AC02	
Oral glucocorticoid	H02AB	
**Diabetes drugs in the last 6 months**		
No diabetes drug	Not any A10	

© 2024 Ueda P et al. *JAMA Intern Med.*

DPP4 inhibitors	A10BH01, A10BH02, A10BH03, A10BH04, A10BH05, A10BD07, A10BD08, A10BD09, A10BD10, A10BD11, A10BD13, A10BD19, A10BD21, A10BD24	
Metformin	A10BA02, A10BD02, A10BD03, A10BD05, A10BD07, A10BD08, A10BD10, A10BD11, A10BD13, A10BD14, A10BD15, A10BD16, A10BD20	
Sulfonylureas	A10BB, A10BD01, A10BD02, A10BD04, A10BD06	
Insulin	A10AB, A10AC, A10AD, A10AE	
Other antidiabetics (glitazones, glinides, acarbose)	A10BF01, A10BG, A10BD03, A10BD04, A10BD05, A10BD06, A10BD09, A10BD14, A10BX	
**Health care utilization in the last year**		
Number of distinct prescription drugs in last year	<5, 6-10, 11-15, ≥16	
Number of outpatient physician visits in last year	0, 1-3, ≥4	
Number of hospital admissions in last year	0, 1-2, ≥3	
Hospital admission within 30 days before index date	N.A.	Patient register, hospitalization

[a] Not included in the propensity score for the secondary outcome analysis of incident depression and anxiety-related disorder as the variables were included in the exclusion criteria for the analysis.
[b] Only available in Denmark.

Abbreviations: glucagon-like peptide 1, GLP-1; sodium-glucose cotransporter 2, SGLT2; ADHD, attention deficit/hyperactivity disorder; DPP4, dipeptidyl peptidase 4

© 2024 Ueda P et al. *JAMA Intern Med.*

eTable 6. Population characteristics for secondary outcome analyses of suicide death and non-fatal self-harm, and self-harm

	Sweden					Denmark				
		Before weighting		After weighting[a]			Before weighting		After weighting[a]	
	GLP-1 receptor agonists	SGLT2 inhibitors	Standardized difference	SGLT2 inhibitors	Standardized difference	GLP-1 receptor agonists	SGLT2 inhibitors	Standardized difference	SGLT2 inhibitors	Standardized difference
N	77439	108838		-		47020	65147		-	
Mean (SD) age	60.3 (12.5)	64.5 (11.1)	35.3	60.2 (10.5)	0.9	58.4 (13.1)	62.7 (11.7)	34.6	58.8 (10.8)	2.9
Women	33434 (43.2)	37040 (34.0)	18.9	43.4	0.5	22246 (47.3)	23561 (36.2)	22.7	46.2	2.1
Men	44005 (56.8)	71798 (66.0)	18.9	56.6	0.5	24774 (52.7)	41586 (63.8)	22.7	53.8	2.1
Place of birth										
Scandinavia	62450 (80.6)	84606 (77.7)	7.2	80.9	0.7	41598 (88.5)	54665 (83.9)	13.2	88.5	0.1
Rest of Europe	5628 (7.3)	9149 (8.4)	4.2	7.2	0.3	2501 (5.3)	4540 (7.0)	6.9	5.4	0.2
Outside Europe	9361 (12.1)	15083 (13.9)	5.3	11.9	0.6	2921 (6.2)	5942 (9.1)	10.9	6.2	0.2
Living with partner										
Yes	37624 (48.6)	56671 (52.1)	7.0	48.2	0.7	29466 (62.7)	41113 (63.1)	0.9	62.4	0.5
No	39815 (51.4)	52167 (47.9)	7.0	51.8	0.7	17554 (37.3)	24034 (36.9)	0.9	37.6	0.5
Education										
Primary school/high school	57804 (74.6)	82557 (75.9)	2.8	75.1	1.0	35900 (76.4)	51417 (78.9)	6.2	77.2	2.0
Vocational or short tertiary education	8592 (11.1)	11574 (10.6)	1.5	10.9	0.5	1695 (3.6)	2230 (3.4)	1.0	3.5	0.6
Medium or long tertiary education	9720 (12.6)	12393 (11.4)	3.6	12.2	1.0	8028 (17.1)	9362 (14.4)	7.4	16.3	2.2
Missing	1323 (1.7)	2314 (2.1)	3.0	1.8	0.5	1397 (3.0)	2138 (3.3)	1.8	3.1	0.6
Calendar year of cohort entry										
2013-2015	12971 (16.7)	6268 (5.8)	35.3	17.7	2.5	8601 (18.3)	5133 (7.9)	31.3	19.6	3.5
2016-2018	24680 (31.9)	32488 (29.8)	4.4	32.2	0.8	10221 (21.7)	20098 (30.9)	20.8	22.5	1.8
2019-2021	39788 (51.4)	70082 (64.4)	26.6	50.1	2.6	28198 (60.0)	39916 (61.3)	2.7	57.9	4.3

© 2024 Ueda P et al. *JAMA Intern Med.*

**History of psychiatric conditions or use of antidepressants**										
Current use of antidepressants (last 6 months)	14666 (18.9)	15956 (14.7)	11.5	19.4	1.2	7664 (16.3)	8192 (12.6)	10.6	16.4	0.4
Previous use of antidepressants (last 10 years but not within 6 months)	25714 (33.2)	29519 (27.1)	13.3	33.9	1.4	14710 (31.3)	16443 (25.2)	13.5	31.5	0.5
Depression or anxiety related disorder within last year	1943 (2.5)	1653 (1.5)	7.1	2.6	0.6	365 (0.8)	386 (0.6)	2.2	0.7	0.5
Previous depression or anxiety-related disorder (at any time before cohort entry, including last year)	9903 (12.8)	9861 (9.1)	12.0	13.1	1.1	2243 (4.8)	2235 (3.4)	6.8	4.8	0.0
Drug misuse within last year	281 (0.4)	262 (0.2)	2.2	0.4	0.5	242 (0.5)	206 (0.3)	3.1	0.6	1.1
Previous drug misuse (at any time before cohort entry, including last year)	1321 (1.7)	1252 (1.2)	4.7	1.9	1.2	699 (1.5)	656 (1.0)	4.3	1.7	1.4
Alcohol related disorders within last year	614 (0.8)	701 (0.6)	1.8	0.9	0.7	380 (0.8)	435 (0.7)	1.6	0.8	0.4
Previous alcohol related disorders (at any time before cohort entry, including last year)	2900 (3.7)	3566 (3.3)	2.5	3.8	0.4	1650 (3.5)	2205 (3.4)	0.7	3.5	0.1
Previous self-harm	1535 (2.0)	1503 (1.4)	4.7	2.1	0.5	76 (0.2)	84 (0.1)	0.9	0.1	0.6
Schizophrenia	1508 (1.9)	1667 (1.5)	3.2	2.0	0.7	610 (1.3)	598 (0.9)	3.6	1.2	0.5
Behavioural syndromes associated with physiological disturbances and physical factors	1056 (1.4)	912 (0.8)	5.0	1.3	0.5	176 (0.4)	161 (0.2)	2.3	0.3	0.5
Disorders of adult personality and behavior	1131 (1.5)	879 (0.8)	6.2	1.6	1.0	272 (0.6)	164 (0.3)	5.1	0.6	0.0
Other psychiatric diagnoses	2140 (2.8)	1776 (1.6)	7.7	2.9	1.0	357 (0.8)	259 (0.4)	4.8	0.8	0.4
Outpatient visit for psychiatric diagnosis during the last year	4256 (5.5)	3847 (3.5)	9.5	5.7	1.0	808 (1.7)	749 (1.1)	4.8	1.6	0.6
Hospitalization for psychiatric diagnosis during the last year	717 (0.9)	731 (0.7)	2.9	1.0	0.3	440 (0.9)	445 (0.7)	2.8	0.9	0.7

© 2024 Ueda P et al. *JAMA Intern Med.*

Outpatient visit for psychiatric diagnosis (but not during last year)	10505 (13.6)	10346 (9.5)	12.7	14.0	1.2	1499 (3.2)	1501 (2.3)	5.4	3.1	0.7
Hospitalization for psychiatric diagnosis (but not during last year)	3625 (4.7)	3728 (3.4)	6.4	4.9	0.8	923 (2.0)	979 (1.5)	3.5	1.9	0.1
**Other medical history**										
Cardiovascular disease	22254 (28.7)	44673 (41.0)	26.0	27.9	1.8	13484 (28.7)	23865 (36.6)	17.0	29.4	1.6
Diabetes complications	28471 (36.8)	37897 (34.8)	4.1	37.3	1.1	14608 (31.1)	19576 (30.0)	2.2	33.5	5.3
Obesity diagnosis	14579 (18.8)	10716 (9.8)	25.8	19.1	0.8	10810 (23.0)	8165 (12.5)	27.6	23.4	0.9
Thyroid disease	1455 (1.9)	1523 (1.4)	3.8	1.9	0.2	1134 (2.4)	1238 (1.9)	3.5	2.4	0.2
**Prescription-drug use in last year**										
Beta-blocker	30278 (39.1)	50409 (46.3)	14.6	38.7	0.9	11649 (24.8)	20505 (31.5)	14.9	25.6	1.9
Opiate	13982 (18.1)	15720 (14.4)	9.8	18.6	1.3	8301 (17.7)	9469 (14.5)	8.5	18.2	1.4
Antipsychotic	2992 (3.9)	2962 (2.7)	6.4	4.1	1.1	2471 (5.3)	2749 (4.2)	4.9	5.4	0.5
Anxiolytic	7256 (9.4)	8436 (7.8)	5.8	9.6	0.8	1966 (4.2)	2347 (3.6)	3.0	4.3	0.7
Hypnotic or sedative	12651 (16.3)	15546 (14.3)	5.7	16.5	0.4	3505 (7.5)	4164 (6.4)	4.2	7.4	0.3
ADHD medications	698 (0.9)	454 (0.4)	6.0	0.9	0.4	402 (0.9)	283 (0.4)	5.3	0.8	0.5
Oral glucocorticoid	7339 (9.5)	9517 (8.7)	2.5	9.6	0.3	2974 (6.3)	3893 (6.0)	1.5	6.4	0.5
**Diabetes drugs in the last 6 months**										
No diabetes drug	8893 (11.5)	14320 (13.2)	5.1	9.8	5.6	9688 (20.6)	8357 (12.8)	21.0	16.3	11.0
DPP4 inhibitors	18999 (24.5)	27256 (25.0)	1.2	26.1	3.6	11453 (24.4)	19576 (30.0)	12.8	26.8	5.6
Metformin	53808 (69.5)	81432 (74.8)	11.9	69.8	0.6	33059 (70.3)	52867 (81.2)	25.5	73.2	6.4
Sulfonylureas	9520 (12.3)	14789 (13.6)	3.9	12.5	0.6	5627 (12.0)	8321 (12.8)	2.4	12.7	2.4
Insulin	30427 (39.3)	23540 (21.6)	39.1	41.4	4.2	9458 (20.1)	6585 (10.1)	28.2	22.3	5.2
Other antidiabetics	3343 (4.3)	4934 (4.5)	1.1	4.4	0.4	150 (0.3)	210 (0.3)	0.1	0.3	0.5
**Health care utilization in last year**										
Number of drugs used										
1 to 5	14475 (18.7)	22857 (21.0)	5.8	17.9	1.9	13308 (28.3)	17814 (27.3)	2.1	26.1	4.9
6 to 10	27900 (36.0)	43522 (40.0)	8.2	35.9	0.2	18805 (40.0)	28443 (43.7)	7.4	40.5	1.0

© 2024 Ueda P et al. *JAMA Intern Med.*

11 to 15	20066 (25.9)	26237 (24.1)	4.2	26.0	0.3	9924 (21.1)	13385 (20.5)	1.4	22.0	2.1
≥16	14998 (19.4)	16222 (14.9)	11.9	20.1	1.8	4983 (10.6)	5505 (8.5)	7.3	11.4	2.7
Number of outpatient physician visits										
0	28622 (37.0)	43532 (40.0)	6.2	37.2	0.5	12215 (26.0)	19943 (30.6)	10.3	25.2	1.8
1 to 3	31171 (40.3)	43158 (39.7)	1.2	40.2	0.1	16478 (35.0)	22281 (34.2)	1.8	35.1	0.2
≥4	17646 (22.8)	22148 (20.3)	5.9	22.6	0.4	18327 (39.0)	22923 (35.2)	7.9	39.7	1.5
Number of hospital admissions										
0	63623 (82.2)	86278 (79.3)	7.3	82.5	1.0	33209 (70.6)	45298 (69.5)	2.4	70.6	0.2
1 to 2	11509 (14.9)	18243 (16.8)	5.2	14.5	1.0	10184 (21.7)	13611 (20.9)	1.9	21.8	0.3
≥3	2307 (3.0)	4317 (4.0)	5.4	3.0	0.1	3627 (7.7)	6238 (9.6)	6.6	7.6	0.2
Hospital admission within 30 days before cohort entry	2589 (3.3)	6317 (5.8)	11.8	3.1	1.6	2805 (6.0)	5174 (7.9)	7.8	5.9	0.1

[a] SMR weighting, in which weights are set to 1 for the GLP1 receptor agonist users (no weighting) whereas the comparator group of SGLT2 inhibitor users is weighted according to propensity score.
Abbreviations: glucagon-like peptide 1, GLP-1; sodium-glucose cotransporter 2, SGLT2; ADHD, attention deficit/hyperactivity disorder; DPP4, dipeptidyl peptidase 4

© 2024 Ueda P et al. *JAMA Intern Med.*

eTable 7. Population characteristics for secondary outcome analysis of incident depression and anxiety-related disorders

	Sweden					Denmark				
		Before weighting		After weighting[a]			Before weighting		After weighting[a]	
	GLP-1 receptor agonists	SGLT2 inhibitors	Standardized difference	SGLT2 inhibitors	Standardized difference	GLP-1 receptor agonists	SGLT2 inhibitors	Standardized difference	SGLT2 inhibitors	Standardized difference
N	44304	68001		44935		28116	43082		28020	
Mean (SD) age	61.4 (12.4)	65.2 (11.1)	31.9	61.3 (9.8)	0.7	59.4 (13.1)	63.5 (11.6)	33.1	59.7 (10.4)	2.4
Women	15988 (36.1)	19972 (29.4)	14.4	36.3	0.4	11716 (41.7)	14026 (32.6)	18.9	40.6	2.2
Men	28316 (63.9)	48029 (70.6)	14.4	63.7	0.4	16400 (58.3)	29056 (67.4)	18.9	59.4	2.2
Place of birth										
Scandinavia	36261 (81.8)	53773 (79.1)	7.0	82.1	0.6	25200 (89.6)	36956 (85.8)	11.7	89.7	0.2
Rest of Europe	2833 (6.4)	4929 (7.2)	3.4	6.3	0.2	1309 (4.7)	2603 (6.0)	6.2	4.7	0.0
Outside Europe	5210 (11.8)	9299 (13.7)	5.8	11.6	0.5	1607 (5.7)	3523 (8.2)	9.7	5.6	0.3
Living with partner										
Yes	23381 (52.8)	38085 (56.0)	6.5	52.5	0.5	18656 (66.4)	28779 (66.8)	0.9	66.2	0.4
No	20923 (47.2)	29916 (44.0)	6.5	47.5	0.5	9460 (33.6)	14303 (33.2)	0.9	33.8	0.4
Education										
Primary school/high school	32764 (74.0)	50837 (74.8)	1.8	74.4	1.0	21096 (75.0)	33659 (78.1)	7.3	76.0	2.2
Vocational or short tertiary education	5090 (11.5)	7389 (10.9)	2.0	11.3	0.6	1053 (3.7)	1515 (3.5)	1.2	3.6	0.5
Medium or long tertiary education	5604 (12.6)	8124 (11.9)	2.1	12.4	0.8	5092 (18.1)	6493 (15.1)	8.2	17.2	2.4
Missing	846 (1.9)	1651 (2.4)	3.6	1.9	0.1	875 (3.1)	1415 (3.3)	1.0	3.2	0.5
Calendar year of cohort entry										
2013-2015	7892 (17.8)	4036 (5.9)	37.4	18.6	2.0	5424 (19.3)	3507 (8.1)	32.8	20.4	2.8
2016-2018	14389 (32.5)	20587 (30.3)	4.7	32.5	0.1	6218 (22.1)	13424 (31.2)	20.6	22.8	1.6
2019-2021	22023 (49.7)	43378 (63.8)	28.7	48.9	1.6	16474 (58.6)	26151 (60.7)	4.3	56.8	3.6
**Other medical history**										
Cardiovascular disease	12089 (27.3)	26079 (38.4)	23.7	26.5	1.8	7529 (26.8)	14934 (34.7)	17.2	27.1	0.7
Diabetes complications	15828 (35.7)	22139 (32.6)	6.7	36.0	0.6	8274 (29.4)	12003 (27.9)	3.5	31.6	4.7

© 2024 Ueda P et al. *JAMA Intern Med.*

Obesity diagnosis	6725 (15.2)	5255 (7.7)	23.6	15.2	0.1	5329 (19.0)	4368 (10.1)	25.2	19.3	0.8
Thyroid disease	655 (1.5)	753 (1.1)	3.3	1.5	0.2	599 (2.1)	709 (1.6)	3.6	2.0	1.0
**Prescription-drug use in last year**										
Beta-blocker	17144 (38.7)	30509 (44.9)	12.5	38.0	1.4	7006 (24.9)	13374 (31.0)	13.7	25.3	0.9
Opiate	5427 (12.2)	6762 (9.9)	7.3	12.5	0.6	3393 (12.1)	4426 (10.3)	5.7	12.2	0.3
Antipsychotic										
Anxiolytic										
Hypnotic or sedative	3010 (6.8)	4685 (6.9)	0.4	6.8	0.1	1033 (3.7)	1510 (3.5)	0.9	3.6	0.2
ADHD medications										
Oral glucocorticoid	3399 (7.7)	5104 (7.5)	0.6	7.8	0.5	1423 (5.1)	2195 (5.1)	0.2	5.0	0.2
**Diabetes drugs in the last 6 months**										
No diabetes drug	4113 (9.3)	8349 (12.3)	9.7	8.3	3.6	5299 (18.8)	5440 (12.6)	17.1	15.1	10.1
DPP4 inhibitors	11496 (25.9)	17377 (25.6)	0.9	27.2	2.8	7404 (26.3)	13472 (31.3)	10.9	28.8	5.6
Metformin	32211 (72.7)	52113 (76.6)	9.0	72.6	0.2	20408 (72.6)	35294 (81.9)	22.4	75.2	5.9
Sulfonylureas	6073 (13.7)	9836 (14.5)	2.2	13.7	0.0	3739 (13.3)	5770 (13.4)	0.3	14.3	2.8
Insulin	17524 (39.6)	13846 (20.4)	42.8	40.9	2.8	5480 (19.5)	3972 (9.2)	29.6	21.3	4.5
Other antidiabetics	2018 (4.6)	3123 (4.6)	0.2	4.7	0.9	100 (0.4)	157 (0.4)	0.1	0.4	0.6
**Health care utilization in last year**										
Number of drugs used										
1 to 5	10690 (24.1)	17931 (26.4)	5.2	23.6	1.3	9700 (34.5)	14096 (32.7)	3.8	32.6	4.0
6 to 10	18078 (40.8)	29832 (43.9)	6.2	41.0	0.4	11848 (42.1)	19618 (45.5)	6.8	43.3	2.3
11 to 15	10369 (23.4)	14271 (21.0)	5.8	23.7	0.7	4932 (17.5)	7288 (16.9)	1.7	18.1	1.3
≥16	5167 (11.7)	5967 (8.8)	9.5	11.7	0.2	1636 (5.8)	2080 (4.8)	4.4	6.1	1.0
Number of outpatient physician visits										
0	19209 (43.4)	30749 (45.2)	3.7	43.9	1.2	8343 (29.7)	14605 (33.9)	9.1	28.9	1.7
1 to 3	17464 (39.4)	26008 (38.2)	2.4	39.2	0.5	10099 (35.9)	14939 (34.7)	2.6	36.2	0.6
≥4	7631 (17.2)	11244 (16.5)	1.8	16.9	1.0	9674 (34.4)	13538 (31.4)	6.4	34.9	1.0
Number of hospital admissions										

© 2024 Ueda P et al. *JAMA Intern Med.*

0	37818 (85.4)	55991 (82.3)	8.2	85.8	1.3	21021 (74.8)	31217 (72.5)	5.2	74.7	0.1
1 to 2	5636 (12.7)	10145 (14.9)	6.4	12.3	1.3	5489 (19.5)	8352 (19.4)	0.3	19.6	0.2
≥3	850 (1.9)	1865 (2.7)	5.5	1.9	0.1	1606 (5.7)	3513 (8.2)	9.6	5.7	0.2
Hospital admission within 30 days before cohort entry	1216 (2.7)	3360 (4.9)	11.4	2.5	1.4	1431 (5.1)	3140 (7.3)	9.1	5.0	0.3

[a] SMR weighting, in which weights are set to 1 for the GLP1 receptor agonist users (no weighting) whereas the comparator group of SGLT2 inhibitor users is weighted according to propensity score.
Abbreviations: glucagon-like peptide 1, GLP-1; sodium-glucose cotransporter 2, SGLT2; ADHD, attention deficit/hyperactivity disorder; DPP4, dipeptidyl peptidase 4

© 2024 Ueda P et al. *JAMA Intern Med.*

eTable 8. Main, additional and sensitivity analyses of the primary outcome of suicide death in Sweden and Denmark

	GLP-1 receptor agonists			SGLT2 inhibitors			
	N	N (%) events	Weighted[a] incidence rate per 1000 person-years	N	N (%) events	Weighted[a] incidence rate per 1000 person-years	Weighted[a] hazard ratio (95% CI)
**Sweden**							
Main analysis	77495	60 (0.08)	0.28	108881	46 (0.04)	0.19	1.44 (0.87-2.37)
Subgroup analyses							
History of psychiatric disorder	33 186	47 (0.14)	0.52	40 795	33 (0.08)	0.37	1.41 (0.79-2.53)
No history of psychiatric disorder	44 304	13 (0.03)	0.10	68 001	13 (0.02)	0.06	1.83 (0.79-4.22)
Additional analyses							
As-treated exposure definition	77 495	31 (0.04)	0.22	108 881	28 (0.03)	0.16	1.44 (0.79-2.64)
Analysis restricted to first year of follow-up	77 495	15 (0.02)	0.22	108 881	18 (0.02)	0.19	1.12 (0.49-2.57)
**Denmark**							
Main analysis	47022	17 (0.04)	0.15	65155	25 (0.04)	0.16	0.94 (0.46-1.91)
Subgroup analyses							
History of psychiatric disorder	18 904	11 (0.06)	0.25	22 048	18 (0.08)	0.26	0.96 (0.41-2.25)
No history of psychiatric disorder	28 116	5 (0.02)	0.07	43 082	7 (0.02)	0.09	0.81 (0.22-3.01)
Additional analyses							
As-treated exposure definition	47 022	10 (0.02)	0.13	65 155	9 (0.01)	0.14	0.88 (0.31-2.49)
Analysis restricted to first year of follow-up	47 022	<5 (0.01)	0.08	65 155	5 (0.01)	0.07	1.06 (0.24-4.69)
Sensitivity analysis							
Adjusting for variable with imbalance after weighting[b]	47 022	17 (0.04)	0.15	65 155	25 (0.04)	0.16	0.94 (0.46-1.91)

[a] SMR weighting using a propensity-score.
[b] Analyses adjusted for the variable of *no diabetes drug in the last 6 months* as imbalance between the groups remained for this variable after propensity-score weighting.

© 2024 Ueda P et al. *JAMA Intern Med.*

eTable 9. Secondary outcome analyses in Sweden and Denmark

	GLP-1 receptor agonists			SGLT2 inhibitors			
	N	N (%) events	Weighted[a] incidence rate per 1000 person-years	N	N (%) events	Weighted[a] incidence rate per 1000 person-years	Weighted[a] hazard ratio (95% CI)
**Sweden**							
Suicide death and non-fatal self-harm	77439	447 (0.58)	2.06	108838	420 (0.39)	2.56	0.81 (0.68-0.95)
Self-harm	77439	393 (0.51)	1.81	108838	383 (0.35)	2.40	0.76 (0.64-0.90)
Incident depression and anxiety-related disorders	44304	3499 (7.9)	28.8	68001	3972 (5.8)	28.2	1.02 (0.96-1.08)
**Denmark**							
Suicide death and non-fatal self-harm	47020	42 (0.09)	0.36	65147	45 (0.07)	0.37	0.99 (0.59-1.67)
Self-harm	47020	26 (0.06)	0.22	65147	21 (0.03)	0.22	1.02 (0.50-2.09)
Incident depression and anxiety-related disorders	28116	1414 (5.0)	20.6	43082	1876 (4.4)	20.5	1.00 (0.92-1.09)

[a] SMR weighting using a propensity-score.

© 2024 Ueda P et al. *JAMA Intern Med.*

eFigure 1. Propensity score distribution in the primary outcome analysis in Sweden

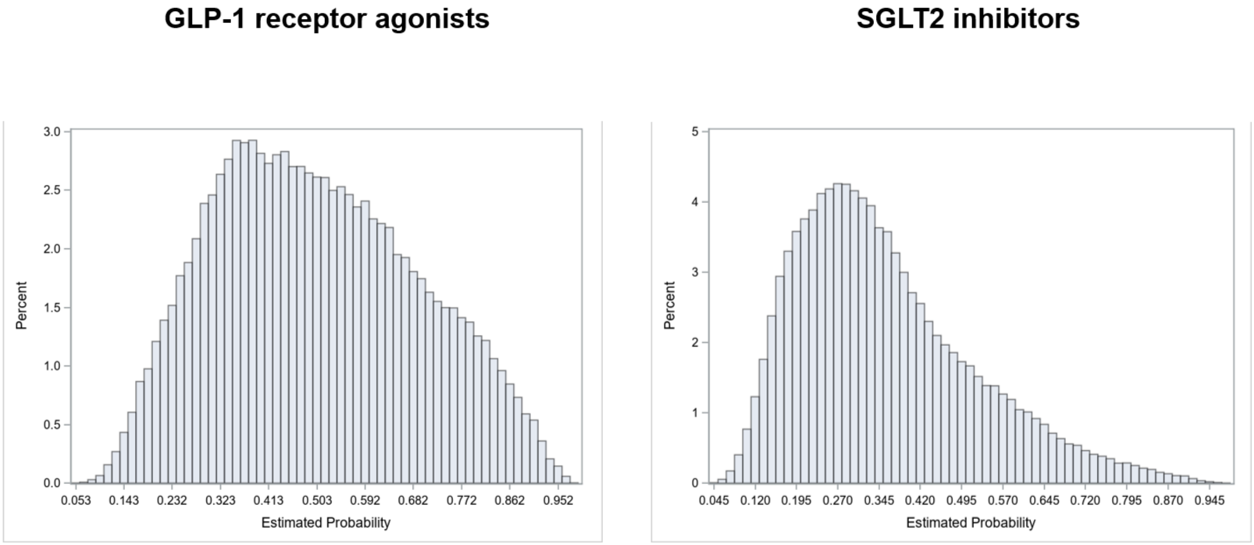

© 2024 Ueda P et al. *JAMA Intern Med.*

eFigure 2. Propensity score distribution in the primary outcome analysis in Denmark

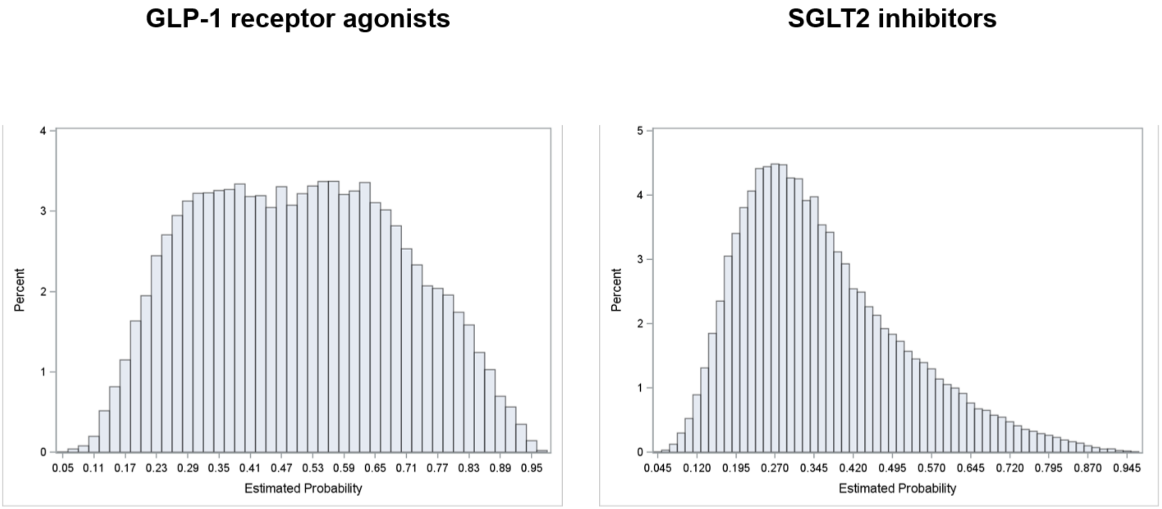

© 2024 Ueda P et al. *JAMA Intern Med.*

eFigure 3. Weighted[a] cumulative incidence for the composite of suicide death and non-fatal self-harm in Sweden (a) and Denmark (b)

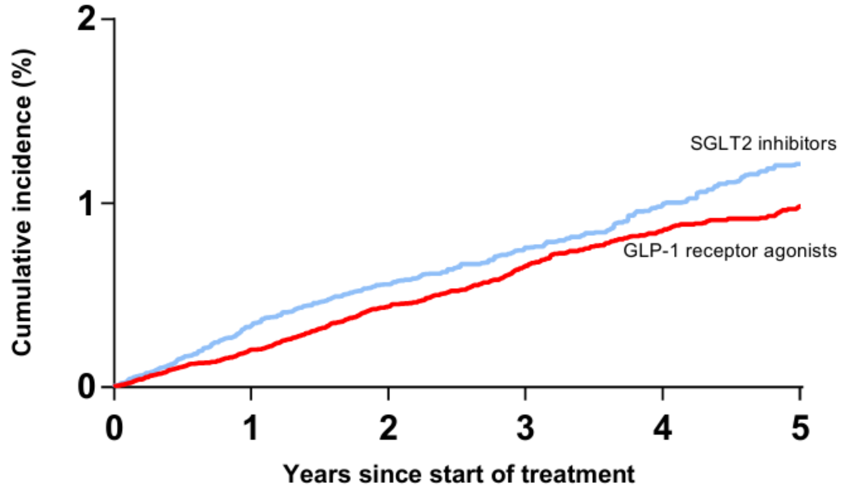

a

Number at risk

GLP-1 receptor agonists	77439	60591	47490	35620	26071	18066
SGLT2 inhibitors	108838	76759	55299	36772	20738	10908

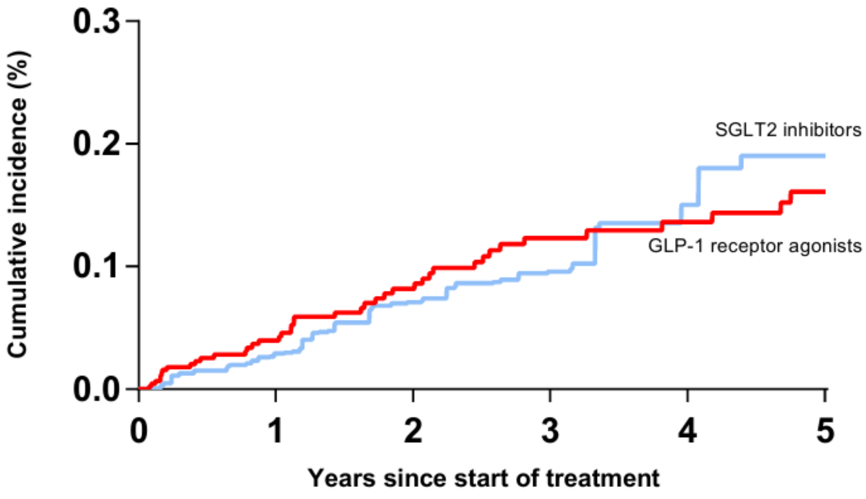

b

Number at risk

GLP-1 receptor agonists	47020	32167	24004	17957	13941	10735
SGLT2 inhibitors	65147	46494	34549	24084	15485	9004

[a] SMR weighting using a propensity score.

© 2024 Ueda P et al. *JAMA Intern Med.*

eFigure 4. Weighted[a] cumulative incidence for self-harm in Sweden (a) and Denmark (b)

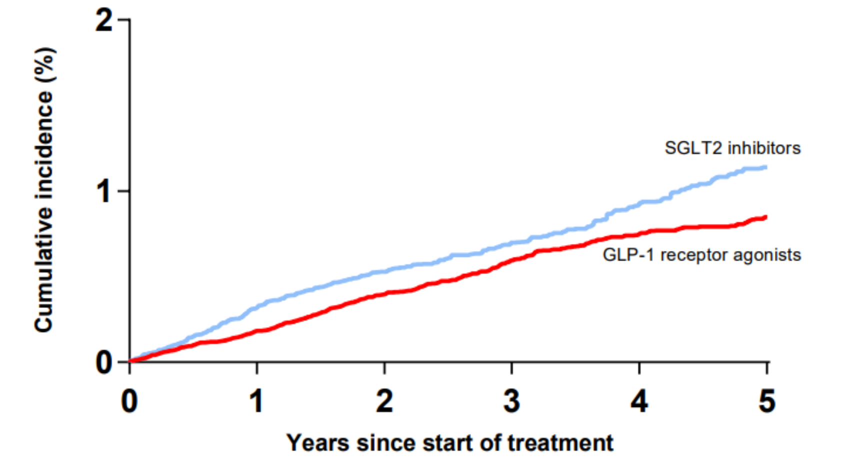

Number at risk

GLP-1 receptor agonists	77439	60591	47490	35620	26071	18066
SGLT2 inhibitors	108838	76759	55299	36772	20738	10908

a

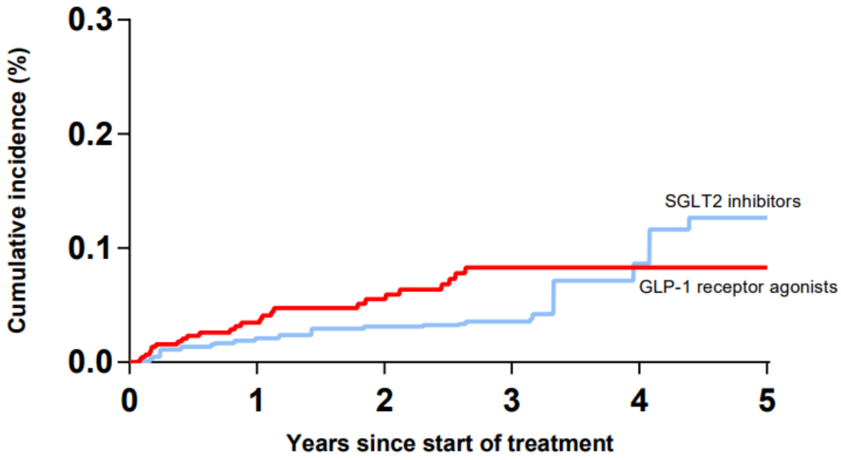

Number at risk

GLP-1 receptor agonists	47020	32167	24004	17957	13941	10735
SGLT2 inhibitors	65147	46494	34549	24084	15485	9004

b

[a] SMR weighting using a propensity score.

© 2024 Ueda P et al. *JAMA Intern Med.*

eFigure 5. Weighted[a] cumulative incidence for the composite of incident depression and anxiety-related disorders in Sweden (a) and Denmark (b)

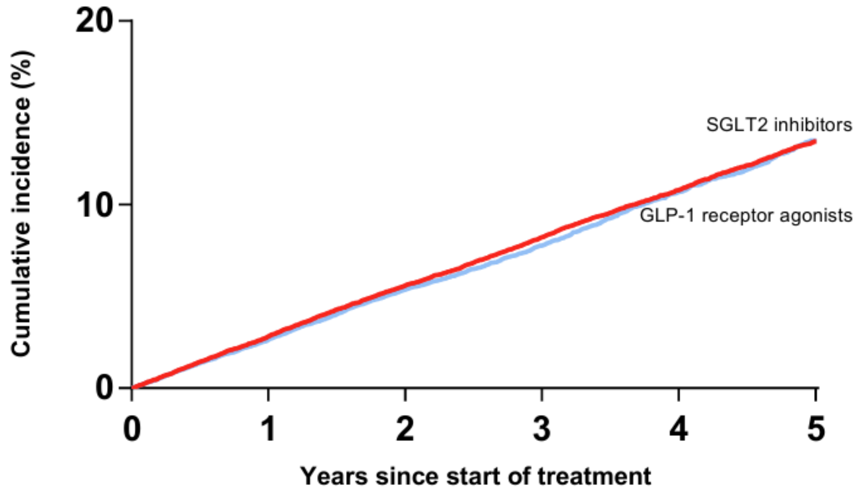

a

Number at risk

GLP-1 receptor agonists	44304	34360	26637	19711	14279	9755
SGLT2 inhibitors	68001	47446	33646	21992	12158	6339

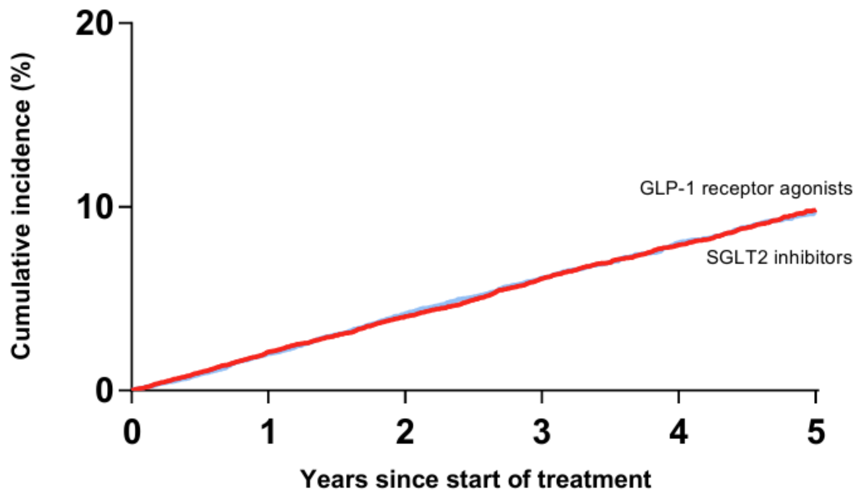

b

Number at risk

GLP-1 receptor agonists	28116	19270	14292	10515	8075	6191
SGLT2 inhibitors	43082	30351	22338	15427	9783	5633

[a] SMR weighting using a propensity score.

© 2024 Ueda P et al. *JAMA Intern Med.*
