# Supplementary material for: GLP-1 Receptor Agonist Use and Risk of Suicide Death
Source: JAMA Intern Med. 2024 Sep 3;184(11):1301–12. doi: 10.1001/jamainternmed.2024.4369 (PMC11372654; doi:10.1001/jamainternmed.2024.4369)
Supplement: Supplement 2. — Data Sharing Statement [file jamainternmed-e244369-s002.pdf]

Data Sharing Statement

Ueda. GLP-1 Receptor Agonist Use and Risk of Suicide Death. *JAMA Intern Med*. Published September 03, 2024. doi:10.1001/jamainternmed.2024.4369

Data
Data available: No

Additional Information
Explanation for why data not available: The data analysed in this study were based on Swedish and Danish nationwide registers. Individual-level data in the registers can only be accessed through secure servers and only export of aggregated data, as presented in research articles, is allowed as per law. Permission to access data can be made only after fulfilling specific requirements to safeguard the anonymity of the study participants. For these reasons, data cannot be made generally available.
